# Structure, function and inhibition of critical protein–protein interactions involving mixed lineage leukemia 1 and its fusion oncoproteins

**DOI:** 10.1186/s13045-021-01057-7

**Published:** 2021-04-06

**Authors:** Xin Li, Yongcheng Song

**Affiliations:** 1 Department of Pharmacology and Chemical Biology, Baylor College of Medicine, 1 Baylor Plaza, Houston, TX 77030 USA; 2 Dan L. Duncan Comprehensive Cancer Center, Baylor College of Medicine, 1 Baylor Plaza, Houston, TX 77030 USA

**Keywords:** Mixed lineage leukemia 1, MLL1-rearranged leukemia, Protein–protein interactions, Protein structure, Drug discovery, Protein inhibition

## Abstract

Mixed lineage leukemia 1 (MLL1, also known as MLL or KMT2A) is an important transcription factor and histone-H3 lysine-4 (H3K4) methyltransferase. It is a master regulator for transcription of important genes (e.g., Hox genes) for embryonic development and hematopoiesis. However, it is largely dispensable in matured cells. Dysregulation of MLL1 leads to overexpression of certain Hox genes and eventually leukemia initiation. Chromosome translocations involving MLL1 cause ~ 75% of acute leukemia in infants and 5–10% in children and adults with a poor prognosis. Targeted therapeutics against oncogenic fusion MLL1 (onco-MLL1) are therefore needed. Onco-MLL1 consists of the N-terminal DNA-interacting domains of MLL1 fused with one of > 70 fusion partners, among which transcription cofactors AF4, AF9 and its paralog ENL, and ELL are the most frequent. Wild-type (WT)- and onco-MLL1 involve numerous protein–protein interactions (PPI), which play critical roles in regulating gene expression in normal physiology and leukemia. Moreover, WT-MLL1 has been found to be essential for MLL1-rearranged (MLL1-r) leukemia. Rigorous studies of such PPIs have been performed and much progress has been achieved in understanding their structures, structure–function relationships and the mechanisms for activating gene transcription as well as leukemic transformation. Inhibition of several critical PPIs by peptides, peptidomimetic or small-molecule compounds has been explored as a therapeutic approach for MLL1-r leukemia. This review summarizes the biological functions, biochemistry, structure and inhibition of the critical PPIs involving MLL1 and its fusion partner proteins. In addition, challenges and perspectives of drug discovery targeting these PPIs for the treatment of MLL1-r leukemia are discussed.

## Introduction

Chromosome translocations involving mixed lineage leukemia 1 (MLL1, also known as MLL or KMT2A) gene located at chromosome 11q23 cause approximately 75% of acute leukemia in infants and 5–10% in children and adults [1], which can be clinically characterized to be acute lymphocytic leukemia (ALL) or acute myeloid leukemia (AML). Unlike other pediatric ALLs (with a 5-year survival of ~ 90%), MLL1-rearranged (MLL1-r) ALL shows a poor prognosis with 5-year survival rates of 34–39% [1–4], while MLL1-r AML patients have similarly poor outcomes to other AMLs with 5-year survival rates of ~ 50% for younger (< 45 years) and < 35% for older patients [5]. In addition, treatment with DNA-topoisomerase II inhibitors poses a risk of 2%–15% to induce MLL1 rearrangement and cause therapy-related secondary leukemia [6, 7]. Current treatments for MLL1-r leukemias are conventional chemotherapeutics, which non-selectively kill all rapidly proliferating cells including normal stem/progenitor cells in the bone marrow and other organs (e.g., intestines). This causes severe toxicities, side effects, and even secondary cancers. Targeted therapeutics against oncogenic fusion MLL1 that drives the malignancy are therefore needed.

**Fig. 1:**
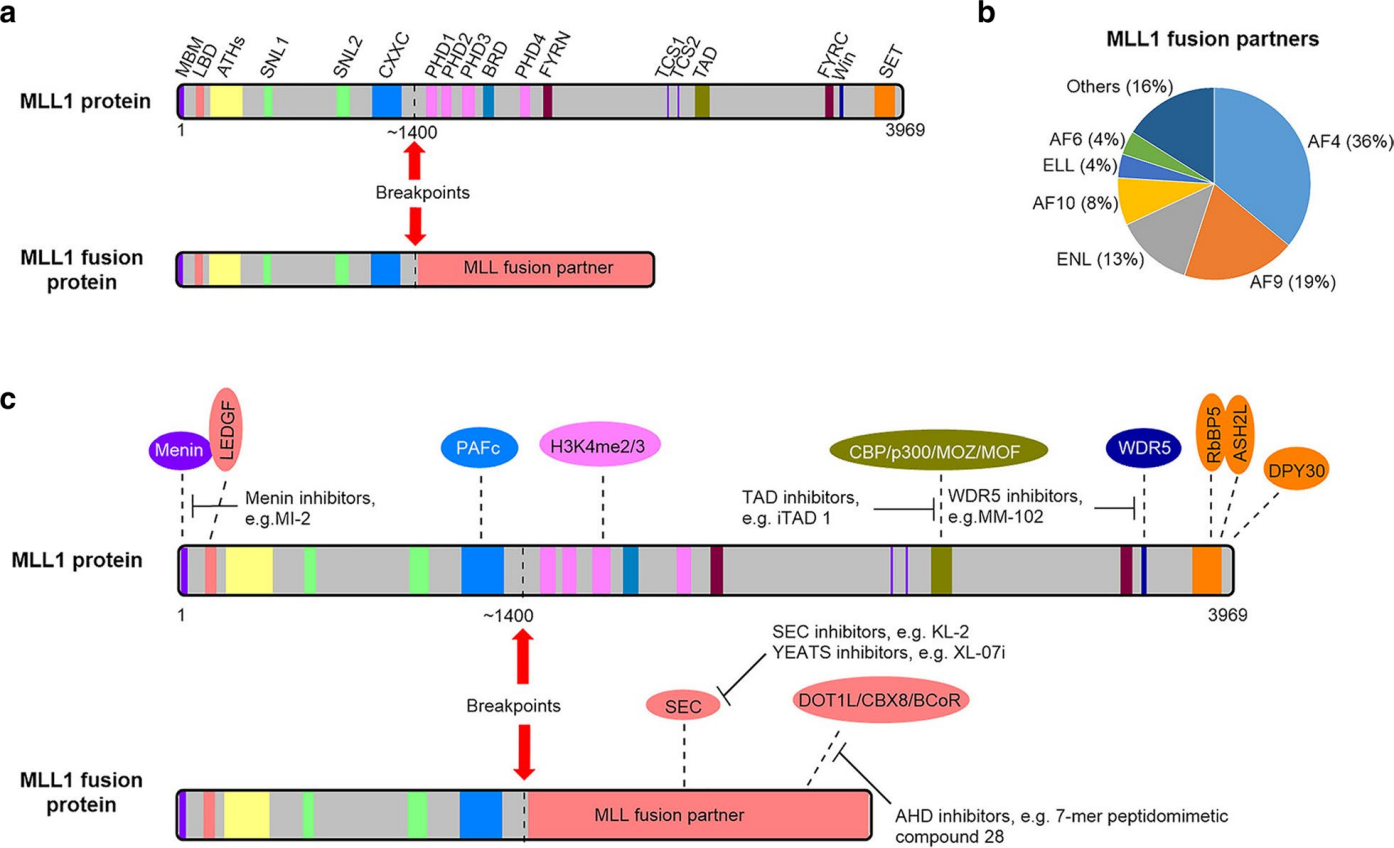
MLL1 and oncogenic MLL1 fusion proteins. **a** Illustrations of MLL1 and onco-MLL1 fusion proteins showing their functional domains. MLL1 with 3969 amino acids contains domains of (from the N- to C-terminus) Menin-binding motif (MBM, residues 2–40), LEDGF-binding domain (LBD, 109–153), AT-hooks (ATHs, 142–400), nuclear-localization signals 1 and 2 (SNL1, 400–443 and SNL2, 1008–1106), CxxC domain (1147–1337), plant homology domains 1–4 (PHD1, 1431–1482; PHD2, 1479–1533; PHD3, 1566–1627; PHD4, 1931–1978), bromodomain (BRD, 1703–1748), FYRN domain (2018–2074), Taspase 1 cleavage sites 1 and 2 (TCS1, 2666–2670 and TCS2, 2718–2722), transactivation domain (TAD, 2829–2883), FYRC domain (3666–3747), WDR5 interaction motif (Win, 3762–3773), and SET domain (3829–2945). The breakpoints of MLL1 are located in the region of ~ 1400. **b** Frequencies of the MLL1 fusion partner proteins in the clinic, with transcription cofactors AF4 (∼36%), AF9 (∼19%) and its paralog ENL (∼13%), AF10 (∼8%), ELL (∼4%) and AF6 (∼4%) being the most common. **c** Illustration of current approaches to inhibit PPIs involving MLL1 and its fusion proteins

First described in 1992 [8, 9], MLL1 is a large, multi-domain protein containing 3,696 amino acid residues (Fig. 1a). Its N-terminal ~ 1,400 residues including AT-hooks (ATH) and CxxC domains act as a transcription factor, recognizing and binding MLL1-target genes, while its C-terminal SET (**S**u(Var)3–9, **e**nhancer-of-zeste, **t**rithorax) domain, a homolog of *Drosophila* trithorax, is a histone-H3 lysine-4 (H3K4) methyltransferase [10]. MLL1 plays crucial roles during early embryonic development and hematopoiesis by regulating the Hox cluster genes expression [11]. In MLL1-r leukemia, chromosome translocation produces an oncogenic fusion protein consisting of the N-terminal DNA-interacting domains of MLL1 (residues 1- ~ 1400) fused with one of > 70 fusion partner proteins (Fig. 1a) [12–14], among which transcription cofactor proteins AF4 (∼36%), AF9 (∼19%) and its paralog ENL (∼13%), AF10 (∼8%), ELL (∼4%) and AF6 (∼4%) are the most frequent [14] (Fig. 1b).

MLL1 as well as its major fusion partners involve numerous protein–protein interactions (PPI), which plays critical roles in regulating gene expression in normal physiology and in leukemia initiation and maintenance. Moreover, the wild-type (WT) MLL1 in the other allele has been found to be essential for MLL1-r leukemia [15]. Rigorous biochemical, biophysical (particularly X-ray crystallography and NMR) and biological studies of such PPIs have been performed and much progress has been achieved in understanding their structures and structure–function relationships as well as the mechanism for leukemogenesis. To a lesser extent, pharmacological inhibition of several critical PPIs has been explored as a targeted therapeutic approach for MLL1-r leukemia, as exemplified by KO-539, an inhibitor of the MLL1-Menin interaction, being in clinical trials [16].

Human MLL1 belongs to the MLL/KMT2 family of lysine methyltransferases (KMT), which includes MLL2 (KMT2D), MLL3 (KMT2C), MLL4 (KMT2B), MLL5 (KMT2E), SET1A (KMT2F), and SET1B (KMT2G). The other MLL1 family proteins are also key regulators for gene transcription and play important roles in normal physiology and diseases [17–19]. This review is focused on MLL1, since it is the most studied and involved in a number of chromosomal translocations causing 5–10% acute leukemia in children and adults with a poor prognosis [10, 20]. We summarize the biology, structures, structure–function relationships and inhibition of the critical PPIs involving MLL1 as well as its major fusion proteins (Fig. 1c).

## Wild-type and oncogenic fusion MLL1

Although classified as a lysine methyltransferase (KMT), MLL1 is mainly a transcription factor and has been found to bind thousands of gene promoters and regulate their expression. It has a global role in the positive regulation of transcription of many important genes in mammals, such as clustered Hox genes which mediate the differentiation of multiple tissues, including the hematopoietic system, during embryogenesis [11, 21, 22]. MLL1 is required during early development and its knockout is embryonic lethal in mice with multiple developmental deficiencies [11]. Conditional MLL1 deletion in mice did not affect the development of mature hematopoietic cells as well as their differentiation, while self-renewal of the stem cells was compromised in fetal liver and adult bone marrow [23]. Moreover, dysregulation of MLL1 leads to constitutive or over-expression of certain Hox genes (e.g., HoxA9), which has been found to cause leukemia [24].

Upon translation, MLL1 is cleaved by the protease Taspase 1 [25], with the N-terminal fragment (MLL1-N) and C-terminal fragment (MLL1-C) forming a protein complex through the interactions between FYRN (Phe/Tyr-rich N-terminal) and FYRC (Phe/Tyr-rich C-terminal) domains [26]. As illustrated in Fig. 1a, MLL1-N contains domains of MBM (menin-binding motifs), LBD (lens epithelium-derived growth factor (LEDGF)-binding domain), ATH (AT-hooks), SNL1 and 2 (nuclear-localization signals 1 and 2), CxxC, PHD1-4 (plant homology domains 1–4), BRD (bromodomain) and FYRN [1]. MBM and LBD can recruit Menin and LEDGF and form a ternary MLL1-Menin-LEDGF complex, which interacts with DNA/chromatin through LEDGF [27]. ATHs bind to the minor groove of AT-rich DNA regions, while CxxC interacts with non-methylated CpG DNA for target gene recognition [28]. The CxxC domains also associate with PAFc (polymerase-associated factor complex) to facilitate MLL1 to recognize its target genes (e.g., HoxA9 and Meis1) [29]. MLL1′s PHD domains mainly recognize di- or tri-methylated H3K4 (H3K4-Me2/3) and facilitate MLL1-mediated gene transcription [30, 31].

MLL1-C consists of a TAD (transactivation domain), FYRC, Win (WD repeat protein 5 (WDR5) interaction motif), and SET domain [1]. TAD can recruit histone acetyltransferases CBP/p300, MOZ and MOF to acetylate histone lysine residues (e.g., H3K27, H3K9 and H4K16) for gene expression activation [32]. The SET domain is a H3K4 methyltransferase, but it is catalytically inactive by itself. Complexation with three other proteins, including WDR5 (WD repeat-containing protein 5), RbBP5 (retinoblastoma binding protein 5), and ASH2L (Set1/Ash2 HMT complex subunit ASH2-like) is required to efficiently methylate H3K4 [1].

Onco-MLL1 proteins contain MLL1 (1– ~ 1400) merged with a variety of fusion partners (Fig. 1a, b). The main function of MLL1 portion is to recognize and bind MLL1-target genes with a high affinity through multivalent interactions involving MLL1-Menin-LEDGF, ATH, and CxxC. This is critical to MLL1-r leukemia, as disruption of any one of these MLL1-DNA/chromatin interactions abrogates onco-MLL1′s capability of leukemogenesis. It is also noted that onco-MLL1 has been found to occupy different gene loci from WT-MLL1 [33] and only regulates a small subset of MLL1-target genes [34]. Moreover, the functions of onco-MLL1 rely on the pre-binding of WT-MLL1 to DNA, which is believed to create an “open” chromatin state and facilitate recruitment of onco-MLL1 as well as its mediated gene expression [15]. Knockdown of WT-MLL1 inhibited aberrant gene expression as well as proliferation of MLL1-r leukemia cells [15, 35, 36]. Arguably, a recent research indicated that MLL2, a homolog of MLL1, plays more important roles in sustaining MLL1-r leukemia through a distinct pathway [37].

Although there are > 70 documented fusion partners of MLL1, transcription cofactors AF4 (also known as AFF1) and its paralog AFF4, AF9 and its paralog ENL, and ELL are found in > 70% MLL1-r leukemias (Fig. 1b) [10, 38]. These proteins have been found to associate with each other in several isolated transcription complexes, which have been commonly called super elongation complexes (SEC) [25, 39, 40]. The biological function of SEC is to release RNA polymerase II (Pol II) from transcription pausing and start transcription elongation. SEC is essential for expression of characteristic genes of MLL-r leukemia (e.g., HoxA9 and Meis1) as well as leukemia transformation. Moreover, SEC was also found to be recruited by MLL1-AF6 and -AF10, two other major fusion partners (Fig. 1b) [25, 40]. Thus, the common feature of these frequent MLL1 fusion partners is their ability to recruit SEC and other associated proteins. Indeed, despite the phenotypic difference (either AML or ALL), MLL1-r leukemias overlap in their gene expression profiles [41], also supporting a common mechanism of leukemogenesis.

## PPIs involving WT-MLL1

MLL1 regulates transcription of critical genes during development. Many proteins have been identified to associate with MLL1 and form a large transcription complex with a molecular mass of ~ 2 million daltons [42]. These proteins play important roles in MLL1-mediated gene regulation. For example, germline knockout of LEDGF, which forms a ternary complex with MLL1 and Menin, is also embryonic lethal in mice showing dysregulated expression of Hox genes [43], suggesting MLL1′s function is dependent on LEDGF. Moreover, formation of such protein complex can stabilize and protect MLL1 from ubiquitination and proteasome-mediated degradation [42]. Table 1 summarizes the binding affinity (dissociation constant *K*_d_), availability of the X-ray/NMR structures and inhibitors of these PPIs.

**Table 1:** Binding affinity (*K*_d_), structure, and inhibitors of the critical PPIs involving MLL1

PPIs	*K*_d_ (μM)	Structures (PDB code)	Inhibitors
Menin–MLL1	0.01 or 0.082 [44, 45]	3U85 [45], 4GQ6 [46]	[16, 46–68]
LEDGF–Menin–MLL1	0.47 or 1.4 [45, 69]	3U88 [45]	[69, 70]
LEDGF–MLL1	14.7 [69]	2MTN [69], 2MSR [70] and 6EMQ [71]	[70]
MLL1(PHD3)-H3K4me3	19 or 30 [31, 72]	3LQJ [30]	None
MLL1(PHD3)-H3K4me2	158 [31]	3LQI [30]	None
MLL1(PHD3-BRD)-H3K4me3	4.3 [30]	3LQJ [30]	None
MLL1(PHD3-BRD)-H3K4me2	6.9 [30]	3LQI [30]	None
MLL1(PHD3)-Cyp33	14.7 [72]	2KU7 [30]	None
MLL1-CBP(KIX)	2.8 or 3.8 [73, 74]	2AGH [75]	[76–78]
MLL1-RbBP5-ASH2L	126 [19]	5F6L [19]	None
MLL3-RbBP5-ASH2L	0.13 [19]	5F6K [19]	None
MLL1-WDR5-RbBP5	None	3P4F [79]	None
MLL1-WDR5	0.12 or 1.7 [80, 81]	3EG6 [81], 3EMH [82], and 4ESG [83]	[36, 81, 84–103]

### Menin–MLL1(1–40) interaction

#### Biological function

Menin, the product of MEN1 gene, contains 610 amino acids [104]. Originally, it was identified as a tumor suppressor in endocrine organs and mutation of Menin resulted in multiple endocrine neoplasia type 1 syndrome [104, 105]. Menin is not homologous to any domains of a known protein [106, 107] and directly interacts with a variety of proteins, showing different functions depending on the context of cell types [108, 109]. Menin is critical for MLL1 to regulate its target genes [110–112]. Menin is also required for MLL1-r leukemia, as conditional knockout of Menin inhibited MLL1-AF9 mediated leukemia transformation and suppressed expression of HoxA9, a characteristic gene for the leukemia. [106, 113]. Menin binds MBM (residues 1–40) of MLL1 (or onco-MLL1) with a high affinity (*K*_d_ = 9.8 nM) and forms a ternary Menin–MLL1-LEDGF complex [44, 45], which greatly enhances MLL1′s ability to recruit LEDGF [69] and tethers MLL1 to chromatin through LEDGF–chromatin interactions (see below). Recent biological studies have shown that Menin’s ability to strengthen the MLL1–LEDGF interaction is critical to MLL-r leukemia. A mutant MLL1(Δ123-153)-ENL, which can bind Menin but not LEDGF, failed to cause leukemia initiation, while an artificial LEDGF(1–93)-MLL1(Δ1-40)-ENL (which cannot recruit Menin), in which the LEDGF(1–93) insert can directly tether MLL1-ENL to chromatin, can cause leukemia transformation [114].

Nonetheless, the Menin–MLL1 interaction is essential for the biological functions of MLL1 and onco-MLL1. There are two Menin-binding motifs in MLL1, termed as MBM1 (residues 2–15) and MBM2 (residues 23–40) [44], with the former having > 20 × higher binding affinity. As for onco-MLL1, disruption of the Menin–MLL1 interaction through deletion of a high-affinity MBM motif (residues 6–10) on MLL1-ENL abolished its leukemia transforming ability in vitro and in vivo [106]. In addition, co-expression of the MLL1(2–44) peptide, which is a dominant negative inhibitor of the Menin–MLL1 interaction, inhibited proliferation of MLL1-AF9 transformed leukemia cells with significantly reduced expression of Meis1 [115]. These studies show that the Menin–MLL1 interaction is a potential drug target for the treatment of MLL1-r leukemia.

#### Structure

**Fig. 2:**
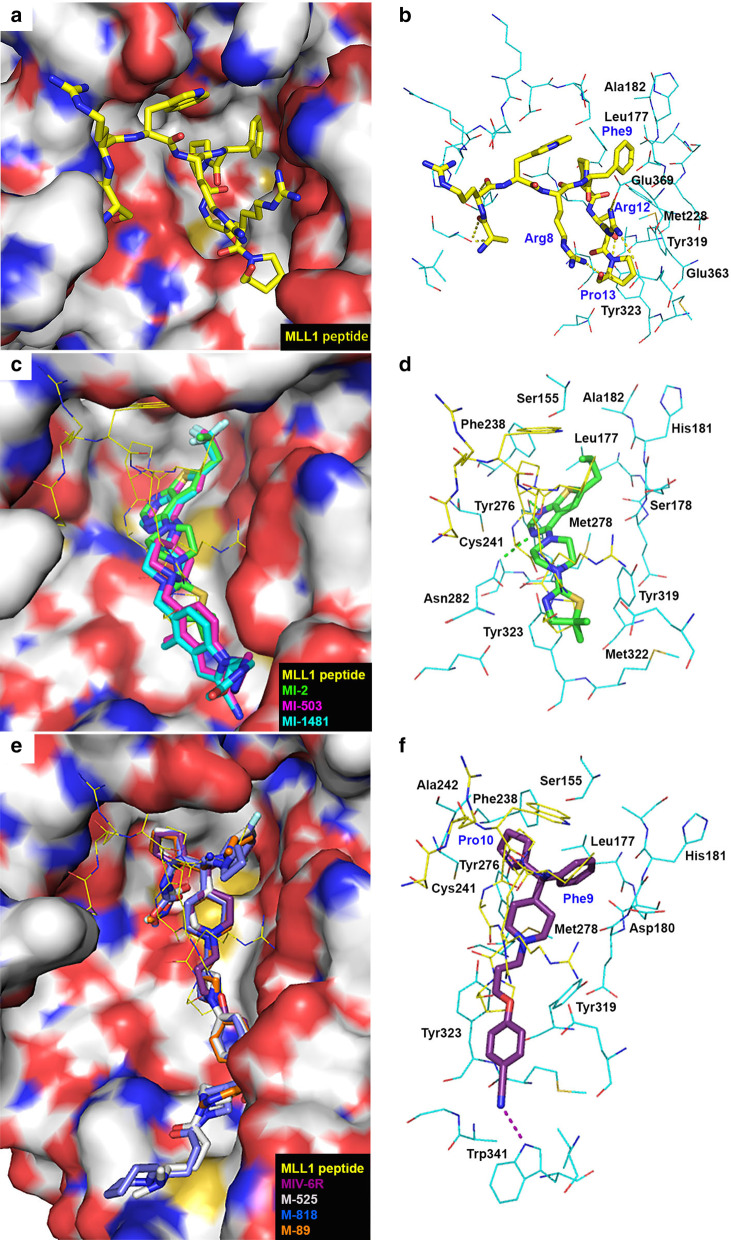
X-ray structures of Menin in complex with MLL1(6–13) peptide and inhibitors. **a** The active site of Menin–MLL1 complex (PDB: 3U85). **b** A close-up view of the Menin–MLL1 interaction. **c** Superimposed active sites of Menin–MLL1, Menin–MI-2 (PDB: 4GQ3), Menin–MI-503 (PDB: 4X5Y), and Menin–MI-1481 (PDB: 6BXY). **d** A close-up view of the Menin–MI-2 interactions. **e** Superimposed active sites of Menin–MLL1, Menin–MIV-6R (PDB: 4OG8), Menin–M-525 (PDB: 6B41), Menin–M-808 (PDB: 6WNH), Menin–M-89 (PDB: 6E1A). **f** A close-up view of the Menin-MIV-6R interactions. MLL1 peptide with C atoms in yellow is shown as a tube model in (**a**) and (**b**), while a line model in (**c**)–(**f**). Compounds MI-2, MI-503, MI-1481, MIV-6R, M-525, M-808, and M-89 are shown as tube models with C atoms in green, magentas, cyan, purple, grey, blue, and orange, respectively. Menin is shown as an electrostatic surface and hydrogen bonds are shown as dashed lines

The crystal structure of Menin in complex with MLL1(6–13) peptide (PDB: 3U85) showed that the MLL1 peptide folds into a bow-shaped conformation and occupies the big central cavity of Menin (Fig. 2a, b) [45, 46]. The sidechain of Arg8 forming an intramolecular hydrogen bond with Pro13 constitutes the “string”. Mutation of either of the two residues significantly decreased the binding affinity. Phe9 occupies a deep hydrophobic cleft formed by Menin residues Leu177, Ala182 and Met228. Mutation of Phe9 with a more polar residue Tyr or His led to > 100-fold affinity reduction. The positively charged Arg12 sidechain inserts into a pocket formed by acidic residues Glu359, Glu363 and Tyr319 of Menin with strong electrostatic and H-bond interactions. A R12A mutation resulted in ~ fourfold loss of binding affinity. Pro13 is located between Menin residues Tyr319 and Tyr323 with favorable hydrophobic interactions. Mutation of either Tyr319 or Tyr323 disrupted these interactions and significantly decreased the binding affinity. The polar and nonpolar interactions account for the high binding affinity of MLL1 to Menin with a *K*_d_ value of 82 nM [45] (Table 1).

#### Inhibitors

**Fig. 3:**
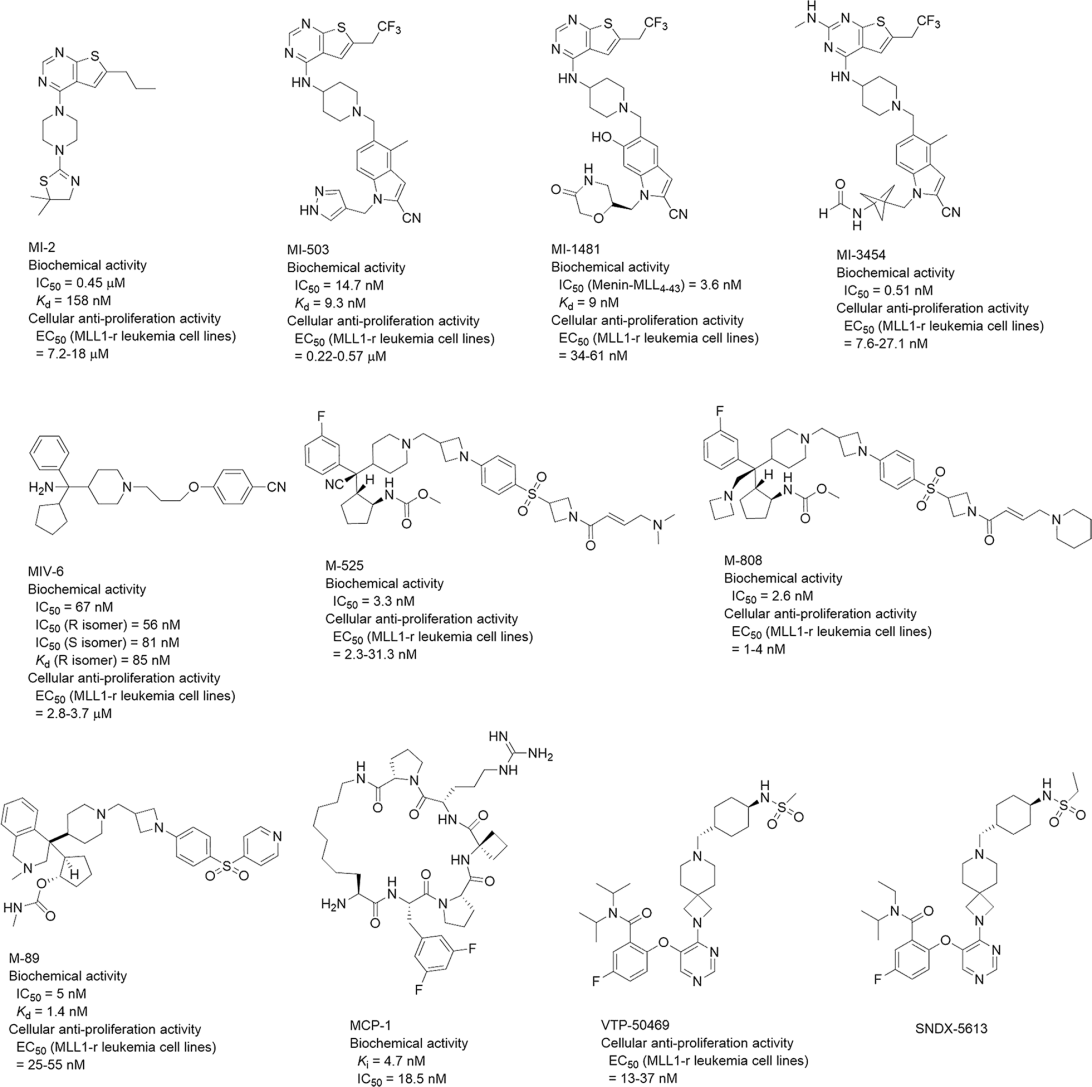
Inhibitors of the Menin–MLL1 interaction

Through high throughput screening (HTS) followed by medicinal chemistry studies, a series of thienopyrimidine compounds have been found to be the first small-molecule inhibitors of the Menin–MLL1 interaction with IC_50_ values as low as 3.6 nM [46, 48–51, 53, 54]. These compounds compete with MLL1 to bind Menin and inhibit the Menin–MLL1 interaction, with the representative compound MI-2 (Fig. 3) exhibiting a *K*_d_ of 158 nM [47]. It also inhibited such interaction in cells, which downregulated expression of the MLL1 target genes, inhibited proliferation (with EC_50_ values of 7–18 µM) and induced hematopoietic differentiation of several MLL1-r leukemia cells. An analog of MI-2 exhibited high synergistic effects when combined with an HDAC inhibitor chidamide against the proliferation of MLL1-r leukemia cells in vitro and in vivo [116]. The crystal structure of Menin in complex with MI-2 (PDB: 4GQ3) shows the inhibitor occupies the binding pocket of the MLL1 peptide (Fig. 2c, d) [46]. The nitrogen atoms of the pyrimidine ring form hydrogen bonds with Tyr276 and Asn282 of Menin. Binding of MI-2 to Menin is further strengthened by favorable hydrophobic interactions between its 6-propyl-thienopyrimidine core structure and Ser155, Leu177, Ser178, His181, Ala182, Phe238, Cys241, Tyr276 and Met278, as well as those between the 4,​5-​dihydro-thiazole fragment of MI-2 and Tyr319, Met322, and Tyr323.

Further optimization of MI-2 yielded the second-generation inhibitors [46, 50–54] with low nanomolar binding affinities to Menin as well as improved cellular activities, such as MI-503 and MI-1481 (Fig. 3). Combination treatment with EPZ004777, an inhibitor of H3K79 methyltransferase DOT1L, had more profound antitumor activity against MLL1-r leukemia [117]. In addition to MLL1-r leukemia cell lines, MI-503 inhibited proliferation of the primary cells from MLL1-r leukemia patients. It was found to have good oral bioavailability, metabolic stability and pharmacokinetics (PK) profiles. MI-503 also exhibited potent in vivo antitumor efficacy in a mouse model of MLL1-r leukemia without apparent toxicity [50, 52]. MI-1481 is the most potent inhibitor in the series with IC_50_ of 3.6 nM, EC_50_ of 34–61 µM as well as strong in vivo antitumor activity [53, 54]. While its oral availability was low, MI-1481 has a high drug exposure in animal plasma. X-ray structures of Menin in complex with MI-503 (PDB: 4X5Y) and MI-1481 (PDB: 6BXY) indicate the inhibitor binds to Menin similarly to MI-2 (Fig. 2c), while their longer 1-substituted-piperidin-4-ylamino groups extending into a side pocket with more favorable hydrophobic and hydrogen bond interactions. This explains their significantly enhanced activities. Further optimization led to more potent analog MI-3454 [55]. BAY-155 is another derivative with low nanomolar inhibitory activity against the Menin–MLL1 interaction as well as MLL1-r leukemia [48, 49].

The second series of inhibitors of the Menin–MLL1 interaction were discovered from HTS followed by medicinal chemistry studies, including MIV-6 (Fig. 3) and its analogs [56–58]. These compounds selectively inhibited the PPI (IC_50_ = 50–90 nM) as well as the proliferation of MLL1-r leukemia cells (EC_50_ = 3–5 µM). They also induced cell differentiation and decreased expression of HoxA9 and Meis1 genes. The crystal structure of Menin in complex with the *R*-enantiomer of MIV-6 (PDB: 4OG8) shows the inhibitor mimics the key interactions between MLL1 and Menin (Fig. 2e, f). The phenyl ring of the compound is located in the binding pocket of MLL1 Phe9, while the cyclopentyl ring fits nicely in that of MLL1 Pro10. The alkoxy linker of the inhibitor mimics the binding of MLL1 Pro13 and the benzonitrile moiety extended into a mostly hydrophobic side pocket, forming a hydrogen bond with Menin residue Trp341.

Systematic optimization of MIV-6 resulted in the discovery of a covalent inhibitor M-525 with an IC_50_ of 3.3 nM [59]. Its X-ray structure (PDB: 6B41) shows M-525 adopts a similar binding mode to MIV-6 (Fig. 2e), while its acrylamide group forms a covalent bond with the Menin residue Cys329, leading to a significantly enhanced binding affinity. Compound M-525 potently inhibited proliferation of MLL1-r leukemia cells with EC_50_ of 2–30 nM, ~ 1000-fold more active than its non-covalent analogs. Another derivative compound M-808 (Fig. 3) [60] showed more potent biological activities than M-525 with a similar binding structure (Fig. 2e). Compound M-808 at a well-tolerated dose was found to cause tumor regression in a leukemia xenograft mouse model. Modification of MIV-6 also yielded a non-covalent inhibitor M-89 with comparable biochemical and biological activities to covalent inhibitor M-525 [61]. Crystallographic studies showed that the overall binding of M-89 (Fig. 2e, PDB: 6E1A) mimics that of MIV-6, while M-89 possesses additional favorable interactions, including hydrogen bond interactions of its carbamate group with Tyr276 as well as more hydrophobic interactions. Treatment with M-89 significantly blocked the growth of MLL1-r leukemia cell lines with low nanomolar EC_50_ values. It also suppressed the expression of MLL1 target genes and induced apoptosis and differentiation of these cells.

There are other inhibitors of the Menin–MLL1 interaction, including macrocyclic peptidomimetic compound MCP-1 [118], a peptidomimetic compound 25 [62], VTP-50469 [63] [64], neomycin [65], tobramycin [65], loperamide [66], DCZ_M123 [67], DC-YM21 [66], and cytisine derivative 1a [68].

Two inhibitors of the MLL1-Menin interaction are in clinical trials (Table 2). Compound KO-539 (IC_50_ = 22 nM, structure undisclosed) potently inhibited proliferation of a panel of MLL1-r leukemia cell lines with EC_50_s of < 25 nM [16]. Treatment with KO-539 significantly reduced tumor burden and prolonged the survivals of mice transplanted with MLL1-r leukemia cells. In addition, treatment with KO-539 also achieved complete remission in mouse models of patient-derived xenograft (PDX) leukemias without overt toxicity. The compound has been in Phase I clinical trial (NCT04067336, https://clinicaltrials.gov/) against relapsed or refractory AML. SNDX-5613 (Fig. 3) [119] has entered a phase 1/2 trial for the treatment of relapsed/refractory leukemias, including those harboring MLL1-r or nucleophosmin 1 (NPM1) mutation (NCT04065399, https://clinicaltrials.gov/).

**Table 2:** Inhibitors targeting PPIs involving MLL1 in clinical trials

Drug	KO-539	SNDX 5613
Tumor type	Advanced malignant neoplasm; Acute myeloid leukemia; Mixed lineage leukemia; Mixed lineage acute leukemia; Acute leukemia of ambiguous lineage; Mixed phenotype acute leukemia	Acute myeloid leukemia; Acute lymphoblastic leukemia; Mixed lineage acute leukemia; Mixed phenotype acute leukemia; Acute leukemia of ambiguous lineage
Phase	Phase 1, Phase 2	Phase 1, Phase 2
Clinical efficacy	Not released	Not released
Safety (% patients)	Not released	Not released
Trial number	NCT04067336	NCT04065399
Status	Recruiting	Recruiting

### LEDGF–MLL1(1–160)-Menin interaction

#### Biological function

**Fig. 4:**
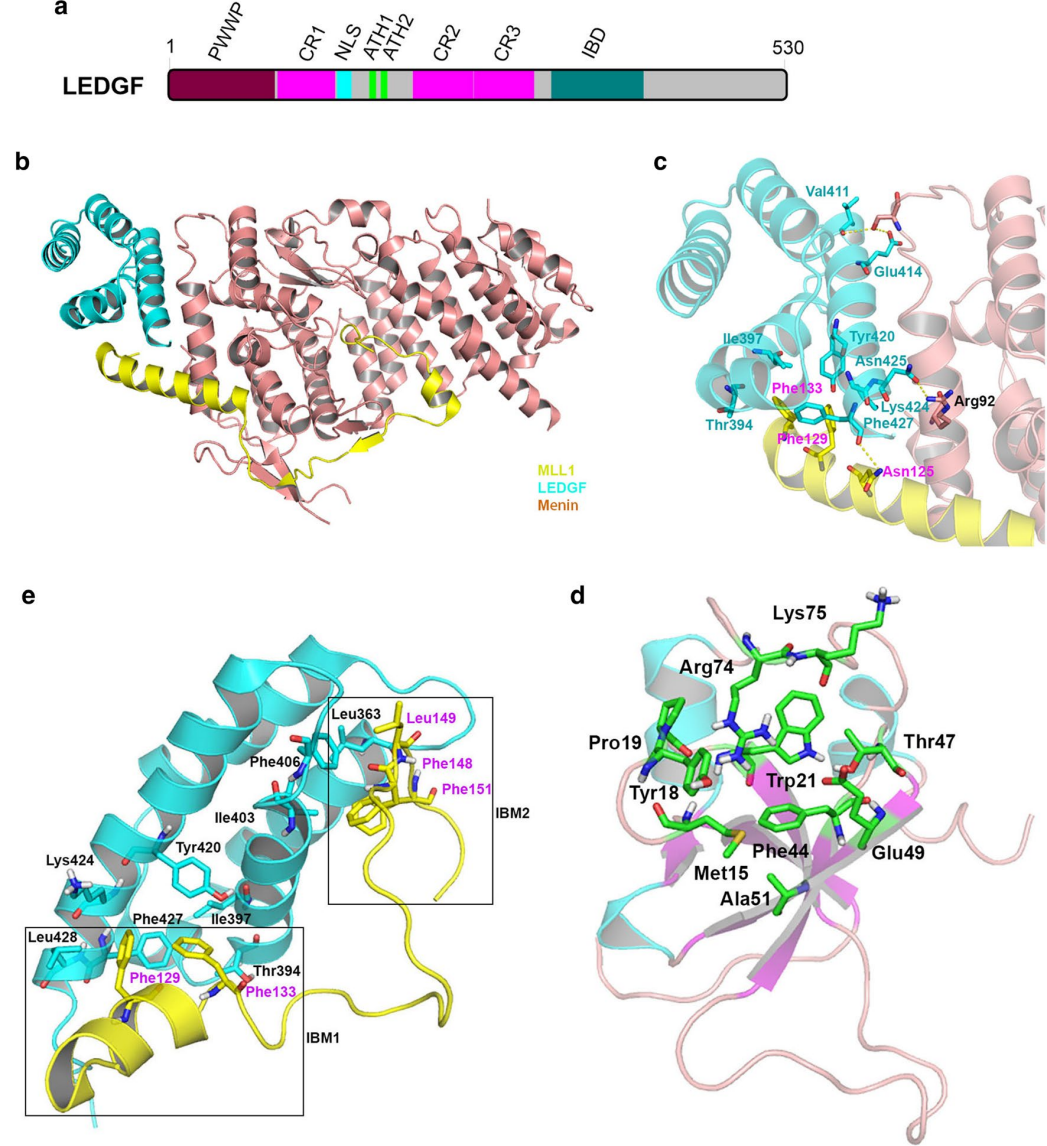
Schematic illustration and structures of LEDGF. **a** Schematic illustration of LEDGF containing a PWWP (residues 1–93), CR1 (charged region 1, residues 106–142), NLS (nuclear localization signal, residues 146–156), ATH1 and 2 (AT-hooks 1 and 2, residues 178–183 and 191–197), CR2 and CR3 (residues 208–266 and 266–325), and IBD (integrase binding domain, residues 347–429) domain. **b**, **c** X-ray structure of LEDGF–Menin–MLL1 complex (PDB: 3U88). The proteins are shown as cartoon with LEDGF in cyan, Menin in orange, and MLL1 in yellow. Overview of the LEDGF–Menin–MLL1 complex in **b** and a close-up view of the interactions between LEDGF and Menin–MLL1 in **c**. The key residues involved in the interaction are shown as stick models. Hydrogen bonds are shown as yellow dashed lines. **d**, **e** NMR structure of LEDGF–MLL1 complex (PDB: 2MTN) and LEDGF PWWP (PDB: 2M16). LEDGF–MLL1 complex with LEDGF in cyan and MLL1 in yellow in **d** and a close-up view of H3K36-Me3 pocket of LEDGF PWWP in **e**. LEDGF PWWP is presented as cartoon with α-helices in cyan, β-sheets in magenta and loops in brown. The key residues involved in the formation of this pocket are shown as stick models with C atoms in yellow

LEDGF/p75, as well as its splice variant p52, was first identified as a transcription coactivator [120]. The full-length LEDGF/p75 consists of 530 amino acids and contains a PWWP (Pro-Try-Try-Pro) domain, AT-hooks (ATH), three charged regions (CR1-3) and an IBD (lentivirus/HIV **i**ntegrase **b**inding **d**omain) domain (Fig. 4a) [121]. Its shorter p52 variant does not have IBD.

LEDGF’s PWWP domain, which is conserved for the protein family, interacts with the chromatin and is essential for the protein function. NMR and other biochemical studies showed that PWWP recognizes and binds mono-nucleosomes containing trimethylated H3K36 (H3K36-Me3) with a high binding affinity (*K*_d_ ~ 100 nM) [27]. PWWP has a well-defined hydrophobic cavity that selectively interacts with H3K36-Me3, while its adjacent basic surface binds DNAs non-specifically. Both events are necessary and cooperative for the tight binding, as PWWP exhibited significantly reduced affinity to either H3K36-Me3 peptide or reconstituted mono-nucleosomes with unmethylated H3K36.

LEDGF’s IBD domain was initially found to bind the integrase of human immunodeficiency virus (HIV) and is required for the function of the integrase and viral replication [123–124]. LEDGF IBD has now been found to bind several other proteins, including MLL1 [45, 114], JPO2, and PogZ [125], and recruit them to the chromatin. Importantly, such PPIs are crucial to the functions of these proteins. For MLL1, LEDGF is indispensable for MLL1-dependent transcription [114]. Knockdown of LEDGF by shRNA decreased colony-forming ability of MLL1-ENL transformed cells with downregulated expression of HoxA9. MLL1-ENL with deleted LBD domain (Δ112-153) or F129A point mutation, which cannot bind to LEDGF with a high affinity, failed to cause leukemic transformation [114].

Further characterization of the MLL1-LEDGF IBD interaction showed that MLL1 has two IBD-binding motifs (IBM) including IBM1 for residues 123–134 and IBM2 for 147–152 [69, 70]. Both are required for the tight binding of LEDGF to MLL1 as well as the biological functions of MLL1/onco-MLL1. Similar to the F129A mutation in IBM1 [114], point mutations of IBM2 (e.g., F148A and L149A) disrupted the MLL1-LEDGF interaction and abrogated leukemogenic transformation of MLL1-AF9 [69, 70]. These and later studies [126] also revealed that LEDGF IBD binds a consensus peptide sequence of D/ExExFxGF in MLL1 IBM2 and several other proteins and such binding is mutually exclusive. These proteins compete with MLL1 to bind to LEDGF IBD and consequently, they are inhibitors of the MLL1-LEDGF interaction. For example, co-expression of HIV integrase or the MLL1(110–160) peptide can disrupt the LEDGF–MLL1 interaction and inhibit proliferation of MLL1-AF9 transformed leukemic cells.

Collectively, high-affinity binding of MLL1 to LEDGF through the MLL1-Menin-LEDGF interactions is essential for MLL1′s biological functions. With its PWWP domain, LEDGF provides an additional anchoring point for MLL1 (in addition to MLL1′s ATH and CxxC domains) to interact with its target genes in the chromatin, which is critical to MLL1′s functions [114]. Deletion of PWWP caused significantly reduced or loss of interaction of LEDGF with the chromatin [121, 127], while substitution of LEDGF PWWP with a PWWP domain from another protein can restore the functions of LEDGF [128–129]. Moreover, while MLL1-ENL without MBM (Menin-binding motif 1–40) cannot cause leukemic transformation, replacement of MBM with LEDGF PWWP (1–93) enabled the engineered MLL-ENL to cause leukemia [114]. However, W21A point mutation in the PWWP of the modified MLL1-ENL failed to do so. These observations show anchoring MLL1 (or onco-MLL1) to the chromatin through its interaction with LEDGF is crucial [114].

#### Structures of the PPI involving LEDGF

The first crystal structure of the Menin–MLL1(6–153)-LEDGF(IBD) ternary complex (PDB: 3U88) was determined at 2.8 Å resolution (Fig. 4b, c) [45]. It is noted MLL1′s IBM2 was not included in the protein complex. LEDGF IBD is bound to a V-shape cleft formed by the Menin N-terminal α-helix and the MLL1 IBM1 α-helix, which is consistent with the observation that LEDGF only strongly interacts with the Menin–MLL1 complex, but not either of them separately [114]. In the MLL1(IBM1)-LEDGF(IBD) interface, LEDGF’s Phe427 forms a hydrogen bond with Asn125 of MLL1. MLL1 residues Phe129 and Phe133 are located in a large hydrophobic pocket of LEDGF consisting of Thr394, Ile397, Tyr420, Lys424 and Phe427. Mutations of the Phe129 and Phe133 to Ala severely disrupted the LEDGF–MLL1 interaction [114]. In the LEDGF–Menin interface, residues Val411 and Glu414 of LEDGF have hydrogen bond interactions with Menin Ser104. The hydrogen bond and electrostatic interactions between LEDGF Asn425 and Menin Arg92 also contribute to their binding. While LEDGF was found not to bind Menin directly, the binding affinity (*K*_d_) of LEDGF to the Menin–MLL1 complex was determined to be 470–1400 nM using isothermal titration calorimetry [45, 69] (Table 1).

NMR structures of LEDGF(IBD) fused with an MLL1 peptide including IBM1 and 2 (PDB: 2MTN, 2MSR, and 6EMQ) were determined in solution [69–71]. The IBM1-LEDGF interaction exhibits a similar binding mode as described in the LEDGF–Menin–MLL1 complex (Fig. 4d). MLL1 IBM1 forms an α-helix, with the key residues Phe129 and Phe133 favorably located in the hydrophobic cavity of LEDGF. For the IBM2–LEDGF interaction which was also found to be critical to biological functions of MLL1/onco-MLL1 [69, 70], the IBM2 peptide is bound in a hydrophobic groove of LEDGF IBD, with Phe148, Leu149 and Phe151 of IBM2 having favorable hydrophobic interactions with the pocket. Mutations of Phe148 and Phe149 of MLL1 abolished the LEDGF–MLL1 interaction in vitro and impaired the leukemic transformation ability of MLL1-AF9 in cells and in vivo. The binding affinity of MLL1 peptide (1–160) to LEDGF was determined with a *K*_d_ of 14.7 μM [69], while complexation with Menin can significantly increase the binding affinity to ~ 1 µM (Table 1) [45, 69].

Since the binding between LEDGF PWWP and the chromatin is critical to MLL1-r leukemia, the NMR structure of LEDGF PWWP (PDB: 2M16) was determined and used to map the binding of H3K36-Me3 and DNA [27]. Similar to other PWWP domains, LEDGF PWWP was found to adopt a characteristic fold with 5 anti-parallel β-strands forming a barrel-shaped core (Fig. 4e), in which there is a hydrophobic cavity, formed by resides Trp21, Phe44, Ala51, Arg74, Lys75, Met15, Tyr18, Pro19, Thr47 and Glu49, to accommodate H3K36-Me3. Adding an H3K36-Me3 peptide, but not unmethylated H3K36 peptide, caused significant changes of the chemical shifts of these residues, indicating H3K36-Me3 interacts with the cavity. In addition, a large adjacent basic surface area is implicated to bind DNA through non-specific electrostatic interactions [27]. Interactions with both H3K36-Me3 and DNA are important and cooperative for the tight binding, as PWWP exhibited significantly reduced affinity to either H3K36-Me3 peptide or reconstituted mono-nucleosomes with unmethylated H3K36.

#### Inhibitors

As the HIV integrase binding site in LEDGF IBD overlaps with that of MLL1 IBM2 and HIV integrase showed > 100-fold enhanced affinity, it was found to be a potent inhibitor (IC_50_ = 301 nM) of the LEDGF–MLL1 interaction [69]. Expression of HIV integrase in MLL-r leukemia cells suppressed the expression of HoxA9 in a dose-dependent manner and exerted antiproliferative activity. Similarly, short MLL1 peptides (residues 146–153, 8-mer) [69] and LEDGF peptides (residues 424–435 and 375–386) [130] were also found to be inhibitors of such PPI. These peptides impaired proliferation of MLL-AF9 transformed leukemic cells. As LEDGF IBD was responsible for the interaction with HIV integrase for the viral replication, there was a significant interest in finding inhibitors targeting the IBD–integrase interaction. Through a phage display strategy, cyclic peptides CP64 and CP65 were found to specifically bind IBD and inhibit the IBD-integrase interaction as well as HIV replication [131]. Later, CP65 was found to also inhibit the MLL1-LEDGF interaction in vitro [70]. In addition, co-expression of CP65 inhibited MLL1-AF9-mediated leukemic transformation and downregulated expression of HoxA9 in these cells.

### PPIs involving MLL1 PHD domains

#### Biological function

PHD domains (~ 50 amino acids) exist in many nuclear proteins in eukaryotic species [132–134] and recognize methylated histone lysine residues for transcription regulation [135–138]. It is characterized by a conserved C4HC2C/H motif that chelate two Zn^2+^ ions [139, 140]. MLL1 contains 4 PHD domains and a bromodomain (BRD) located in between PHD3 and 4. The biological functions of PHD1, 2 and 4 are not well understood. PHD1 and 4 were found to be involved in intramolecular interactions between MLL1-N and -C [140], while PHD2 plays a role in ubiquitination and proteasome-mediated degradation of MLL1 [141].

MLL1 PHD3 domain is most studied and characterized. It recognizes and binds H3K4-Me2 or 3, which is known to be a histone mark for active gene transcription. While the adjacent BRD was found not to recognize acetylated histone lysine residues (as other bromodomain proteins), it can enhance the binding affinity of PHD3 to H3K4-Me3 by ~ sevenfold from a *K*_d_ value of ~ 30 μM to 4.3 μM (Table 1) [30, 31, 72]. PHD3 has been found to facilitate MLL1 to bind at the HoxA9 gene locus and maintain active transcription of the gene [15, 31]. W1594A or H1596A mutation of PHD3 with impaired binding to H3K4-Me3 caused loss of localization of MLL1 at the HoxA9 and Meis1 loci, as well as suppressed expression of these MLL1-target genes [31]. However, M1585A of PHD3 led to reduced binding to H3K4-Me3 and lowered expression of HoxA9 and Meis1, but it did not affect MLL1′s binding to the two genes. These results suggest MLL1 PHD3′s ability to bind H3K4-Me3 might not affect MLL1′s localization in the genome, but it seems to be critical to the expression of MLL1 target genes.

In addition, PHD3 also interacts with the RNA recognition motif (RRM) of the nuclear cyclophilin Cyp33 [142], a transcription co-repressor protein which recruits histone deacetylase 1 (HDAC1) and suppresses the expression of MLL1 target genes [142, 143]. Moreover, the binding of Cyp33 to PHD3-BRD inhibits their interaction with H3K4-Me3 [72]. In addition to its RRM, Cyp33 contains a C-terminal peptidyl prolyl isomerase domain [144], which catalyzes the *cis*–*trans* isomerization of Pro1629 in the linker region of PHD3-BRD and such conformational change disrupts the PHD3-BRD interaction and unblocks the PHD3-Cyp33 RRM interaction [30]. Thus, the *cis*–*trans* isomerization of MLL1′s Pro1629 appears to act as a gene expression switch. The *cis*-configuration favors BRD-PHD3-H3K4-Me3 which turns on MLL1-target gene transcription, while *trans*-Pro1629 enables PHD3 to recruit Cyp33 and suppresses the gene expression. Biologically, overexpression of Cyp33 decreased the levels of H3K4-Me3 at MLL1 target gene loci and downregulated MLL1-mediated gene transcription [72]. No inhibitors have been reported to disrupt the PPIs between PHD3-BRD and H3K4-Me3 or Cyp33.

PHD domains are lost for all oncogenic fusion MLL1. In addition, inclusion of PHD3 in MLL1-ENL abrogated the leukemic transformation ability of the oncogene [145, 146]. These results suggest loss of PHD domains seems to be required for MLL1-r leukemia.

#### Structures involving MLL1 PHD3

**Fig. 5:**
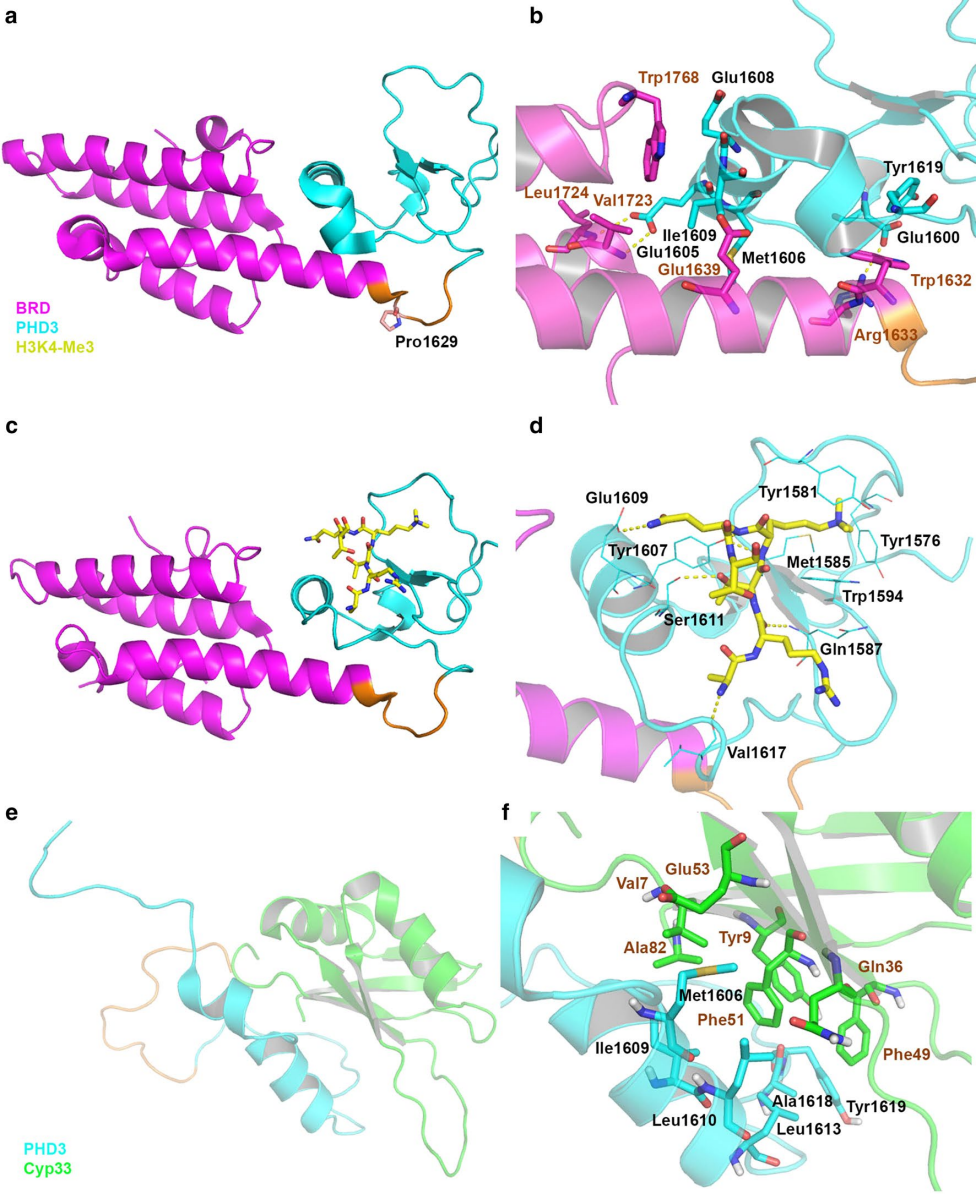
Structure of PHD3-BRD and Cyp33-MLL1(PHD3) complex. **a**–**d** X-ray structures of the PHD3-BRD protein complexes. PHD3 is shown in cyan, BRD in magenta and the flexible loop in between the two domains in orange. Overview of apo-PHD3-BRD (PDB: 3LQH) in (**a)**. A close-up view of the PHD3-BRD interaction in **b**. Overview of the PHD3-BRD-H3K4-Me3 complex (PBD: 3LQJ) in **c**. A close-up view of the PHD3-H3K4-Me3 interaction in **d**. The H3K4-Me3 peptide is shown as a stick model with C atoms in yellow. Hydrogen bonds are shown as yellow dashed lines. **e**, **f** Solution NMR structure of the Cyp33-MLL1(PHD3) complex with PHD3 in cyan, Cyp33 in green and the linker between them in orange (PDB: 2KU7). Overview of Cyp33-MLL1(PHD3) complex in **e**, and a close-up view of Cyp33-MLL1(PHD3) interaction in **f**

The crystal structures of MLL1 PHD3-BRD in apo-form (PDB: 3LQH) as well as in complex with H3K4-Me2 and 3 peptides (PBD: 3LQI and 3LQJ) were determined [30]. In the apo-structure (Fig. 5a, b), a flexible loop (residues 1625–1631, in orange) links the structured PHD3 and BRD domains, both of which adopt a characteristic fold of their family proteins [147]. Pro1629 in *cis*-conformation serves as a crucial turn point, facilitating the PHD3-BRD interaction and inhibiting that of PHD3-Cyp33. For the PHD3-BRD interaction, Glu1605 in PHD3 interacts with Leu1724 and Val1723 in BRD through two hydrogen bonds and Glu1600 in PHD3 forms another hydrogen bond with Arg1633 in BRD. In addition, there are favorable hydrophobic interactions between Met1606, Glu1608, Ile1609, and Tyr1619 in PHD3 and Trp1632, Glu1639 and Trp1768 in BRD.

There are no major conformational changes when the H3K4-Me3 peptide binds to PHD3-BRD, except that the loop consisting of the residues 1612–1618 is moved slightly to widen the binding groove for the H3K4-Me3 peptide. The peptide forms a β-turn and interacts with PHD3. Without interactions with the H3K4-Me3 peptide, BRD can enhance the binding affinity of the peptide to PHD3 by ~ 20-fold, presumably through its interactions with PHD3 [30, 31, 72]. The trimethylated lysine sidechain is inserted into an aromatic cage formed by Try1576, Try1581, Met1585 and Trp1594 of PHD3. It is of interest that as compared to the apo-structure, the orientations of these residue sidechains are changed significantly to accommodate H3K4-Me3. The peptide form hydrogen bond and electrostatic interactions with Met1585, Gln1587, Try1607, Glu1608, Ser1611, and Val1617 of PHD3. The H3K4-Me2 peptide exhibits almost the same binding mode to PHD3-BRD. Mutagenesis studies confirmed the importance of the residues Tyr1581, Gln1587, and Trp1594 in the interactions between PHD3-BRD and H3K4-Me2/3. Consistent with structure analysis, H3K4-Me2/3 show similar binding affinity to PHD3-BRD in ITC assay with *K*_d_ values of 6.9 and 4.3 μM [30] (Table 1), respectively.

As described earlier, Cyp33′s peptidyl prolyl isomerase domain [144] catalyzes the *cis*–*trans* isomerization of Pro1629, which disrupts the PHD3-BRD interaction and the released PHD3 interacts with Cyp33′s RRM domain [30]. The PHD3-Cyp33 binding recruits HDAC1 and suppresses the expression of MLL1 target genes [142, 143]. An NMR structure of Cyp33(RRM) in complex with MLL1(PHD3) (Fig. 7e/f, PDB: 2KU7) [30] shows that formation of the RRM-PHD3 complex is mostly driven by hydrophobic interactions. The sidechains of Met1606, Ile1609, and Leu1610 of PHD3 occupy a hydrophobic groove formed by the RRM residues Val7, Gln36, Phe51, Glu53 and Ala82. There are also favorable interactions between Leu1613, Ala1618 and Tyr1619 of PHD3 and Tyr9 and Phe49 of RRM. The binding affinity (*K*_d_) of the PPI was determined to be 14.7 μΜ [72].

### MLL1(TAD)–CBP(KIX) interaction

Human CBP (**c**AMP-response element binding protein (CREB)-**b**inding **p**rotein), as well as its paralog p300 (E1A-associated protein p300), is a multidomain transcription coactivator containing a histone acetyltransferase (HAT) domain. Through acetylation at various histone lysine residues, CBP/p300 play important roles in active gene transcription [148, 149]. Moreover, CBP/p300 can acetylate a number of transcription factors to regulate gene expression [150, 151]. In addition to its HAT activity, CBP/p300 also serves as a hub protein for the assembly of transcriptional protein complexes [148]. The KIX domain of CBP/p300 mediates PPI with a number of transcription factors, including MLL1, CREB, and c-Myb [152, 153]. The MLL1(TAD)–CBP(KIX) interaction has been found to be critical to MLL1-mediated gene expression [73]. Moreover, the MLL1–CBP interaction can enhance the interaction between CBP and c-Myb [73, 154].

**Fig. 6:**
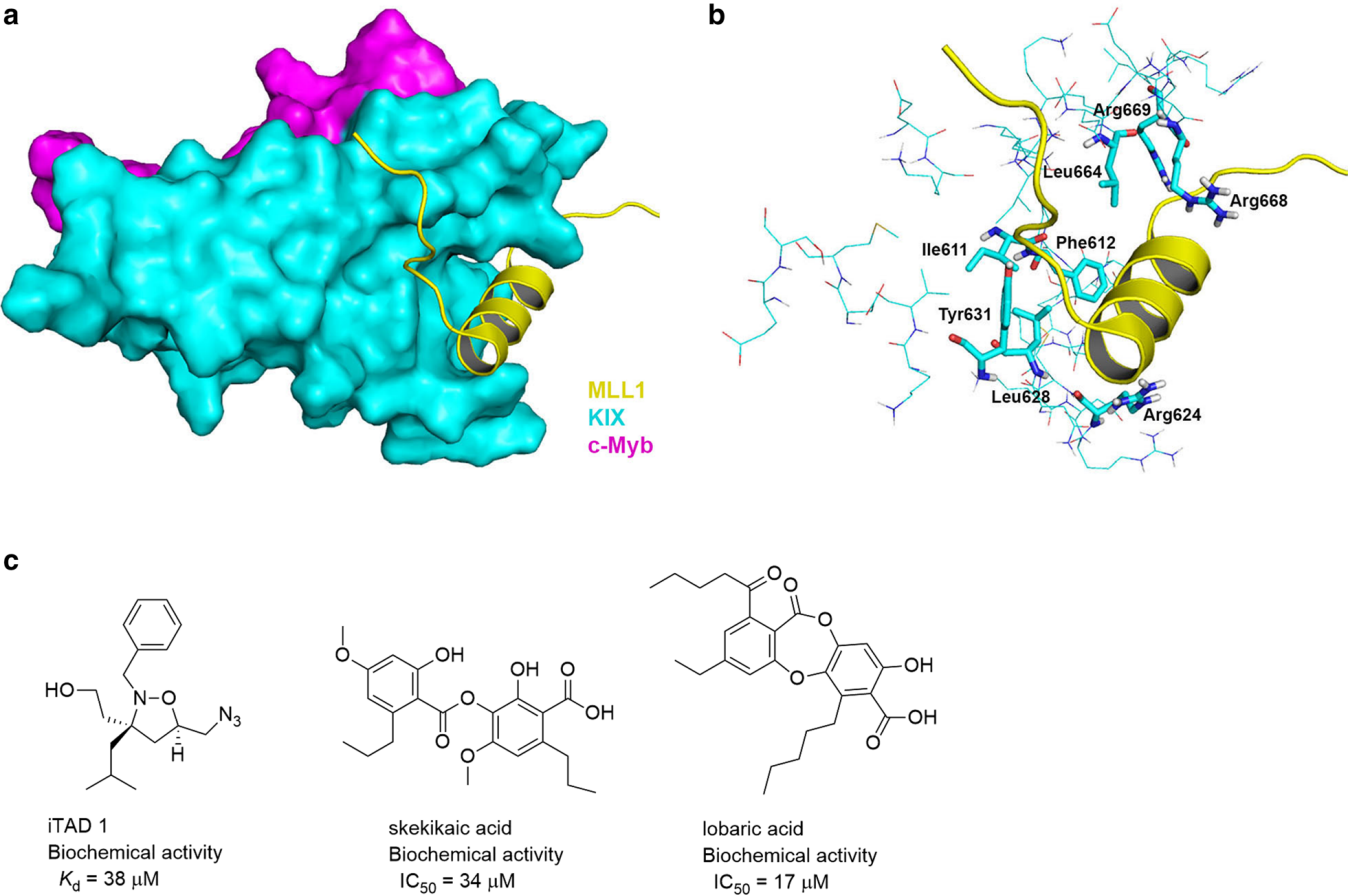
NMR structure of KIX-c-Myb-MLL1 ternary complex (PDB: 2AGH) and compounds inhibiting the MLL1-CBP interaction. **a** Overview of MLL1 peptide binding to KIX-c-Myb complex. MLL1 is shown as sticks with C atom in yellow. KIX and c-Myb are shown as surfaces in cyan and magenta, respectively. **b** A close-up view of the interactions between MLL1 and KIX-c-Myb complex. **c** compounds inhibiting the MLL1-CBP interaction

The solution structure of an MLL1(TAD)-CBP(KIX)-c-Myb ternary complex (PDB: 2AGH) was determined using NMR [75]. MLL1 (residues 2844–2857) forms an α-helix, occupies a large groove of the KIX domain of CBP, and has no contact with c-Myb (Fig. 6a). MLL1′s binding is mostly driven by hydrophobic interactions with the groove of KIX formed by Ile611, Phe612, Arg624, Leu628, Tyr631, Leu664, Arg668, and 669 (Fig. 6b). A mutagenesis study confirmed the importance of Leu628 to the PPI [155]. The binding affinity (*K*_d_) was determined to be ~ 3 μM [73, 74] (Table 1).

In addition to a MLL1-mimetic peptide inhibitor [78], a series of isoxazolidines were found to bind the KIX domain of CBP and inhibit the MLL1-CBP interaction [76]. The representative compound iTAD 1 exhibited a modest IC_50_ of 38 µM (Fig. 6c). In addition, sekikaic and lobaric acid were also identified to be weak inhibitors of the PPI [77].

### PPIs involving MLL1′s H3K4 methyltransferase SET domain

#### Biological function

The SET domain of human MLL1 (residues 3829–3945) is highly conserved during evolution, which was first found in *Drosophila* proteins Su(Var)3–9, Enhancer-of-zeste and Trithorax [156]. Based on this homology, MLL1 has been classified into a family of histone/protein lysine methyltransferases (KMT) in mammals, which include MLL2-5 and SET1A and B. In complexation with several proteins, MLL1′s SET catalyzes mono-, di-, and tri-methylation of H3K4 [157]. Because methylated H3K4 has been linked to active gene transcription [158], MLL1 SET as well as its mediated H3K4 methylation was believed to be essential for MLL1 to regulate gene expression. However, although germline knockout of MLL1 is embryonic lethal [11], mouse embryos bearing homozygous MLL1 with SET truncated (MLL1(ΔSET)) can develop and grow into adulthood without apparent weight or growth differences, except that there were minor skeletal defects and altered gene expression associated with reduced H3K4 mono-methylation at certain Hox gene loci [159]. Global H3K4 methylation was not significantly affected by the SET deletion. More investigation showed that the hematopoietic stem/progenitor cells (which are particularly sensitive to the loss of MLL1) of these MLL1(ΔSET) mice had normal hematopoiesis as well as expression of MLL1 target genes [160]. These mouse model and cell-based studies support that MLL1 SET is largely dispensable for MLL1-regulated hematopoiesis. Moreover, MLL1-AF9 was found to cause leukemic transformation of the MLL1(ΔSET) hematopoietic stem/progenitor cells without significant differences from those with WT MLL1 [160], indicating SET is also dispensable for onco-MLL1-mediated leukemogenesis. Collectively, SET as well as its H3K4 methylation activity is not responsible for MLL1′s major biological functions and more studies of MLL1 SET are therefore needed.

Nonetheless, biochemistry and structural biology of the H3K4 methyltransferase of MLL1 have been well studied. All the MLL family KMTs exhibit a similar mechanism of action. Their SET domain itself is catalytically inactive. Complexation with RbBP5 and ASH2L is required for SET to become an active methyltransferase [19]. Moreover, different from other MLL family proteins, MLL1 does not bind to RbBP5-ASH2L strongly (with *K*_d_ ~ 126 µM, versus *K*_d_ ~ 130 nM for MLL3). Therefore, MLL1-RbBP5-ASH2L exhibited only weak activity. Further inclusion of WDR5 can significantly enhance the catalytic activity [19], because WDR5 binds both MLL1 and RbBP5 to link the two proteins. In contrast, the ternary complexes of MLL2-4, SET1A or B with RbBP5 and ASH2L are fully active and WDR5 is unnecessary. These proteins showed a high binding affinity to the RbBP5-ASH2L complex in pull-down and fluorescence polarization assays, with MLL3 being the strongest.

The structural basis of these PPIs has been investigated. X-ray structures of MLL3 and N3861I/Q3867L mutant MLL1 (which has a high affinity to RbBP5-ASH2L) in complex with RbBP5-ASH2L, including a SAM analog *S*-adenosyl-*L*-homocysteine and the substrate histone H3(1–9) peptide (PDB: 5F6K and 5F6L), were determined and reveal the PPIs among these proteins [19]. The C-terminal SPRY domain of ASH2L interacts with the ASH2L binding motif (ABM) of RbBP5 [161, 162]. RbBP5 also has a WDR5-binding motif (WBM) which can bind and recruit WDR5. For MLL1, structural analysis showed that the hydrophilicity and/or bulkiness of Asn3861 and Gln3867 (as contrast to Ile/Leu or Thr/Val in MLL2 or 3) significantly weaken MLL1′s affinity to RbBP5-ASH2L, such that WDR5 is required for MLL1 to strongly associate with two cofactor proteins for H3K4 methylation [19]. A double-mutant MLL1 with N3861I and Q3867L behaved similarly to MLL3 and WDR5 is unnecessary for its catalytic activity. Mechanistically, the SET domain of the MLL family proteins alone was thought to adopt an open conformation that does not allow an efficient methyl transfer from SAM to the amino group of the lysine sidechain [163]. However, it was later found that upon binding with RbBP5-ASH2L, the active enzyme exhibits no conformational changes [19]. Rather, the conformation of the MLL family SET is highly dynamic and it cannot efficiently perform the catalytic reaction. Complexation with RbBP5-ASH2L significantly reduces the flexibility and enhances the MLL family proteins’ ability to bind SAM and recognize the substrate.

Given WDR5′s critical role in MLL1-catalyzed H3K4 methylation, the WDR5-MLL1 interaction has been investigated as a potential target for intervention. In addition, since WDR5 is only essential for MLL1 SET, targeting WDR5-MLL1 could achieve a high selectivity over other MLL family methyltransferases. Knockdown of WDR5 was found to decrease cellular H3K4me3 levels on HoxA9 and HoxC8 loci and suppressed the expression of these genes [164]. The residues 3745–3969 in MLL1 was identified to be the Win using sedimentation velocity analysis, with the key residue Arg3765 bound to the arginine binding pocket of WDR5, which was previously found for the recognition of histone H3K4me2 [80, 82]. Substitution of Arg3765 with an alanine or mutation of the residues in the arginine binding pocket of WDR5 disrupted the PPI [80]. A Win-derived peptide can dose-dependently inhibit the WDR5-MLL1 interaction and prevent MLL1 from association with WDR5-RbBP5-ASH2L, thereby inhibiting the catalytic activity of MLL1. Thus, disruption of WDR5-MLL1 interaction can selectively inhibit MLL1′s catalytic activity in vitro, although as described above, more convincing evidence is needed to clarify whether targeting such PPI as well as MLL1-catalyzed H3K4 methylation affects MLL1-mediated hematopoiesis and MLL1-r leukemia.

#### Structures of the MLL1-WDR5-RbBP5 complex

**Fig. 7:**
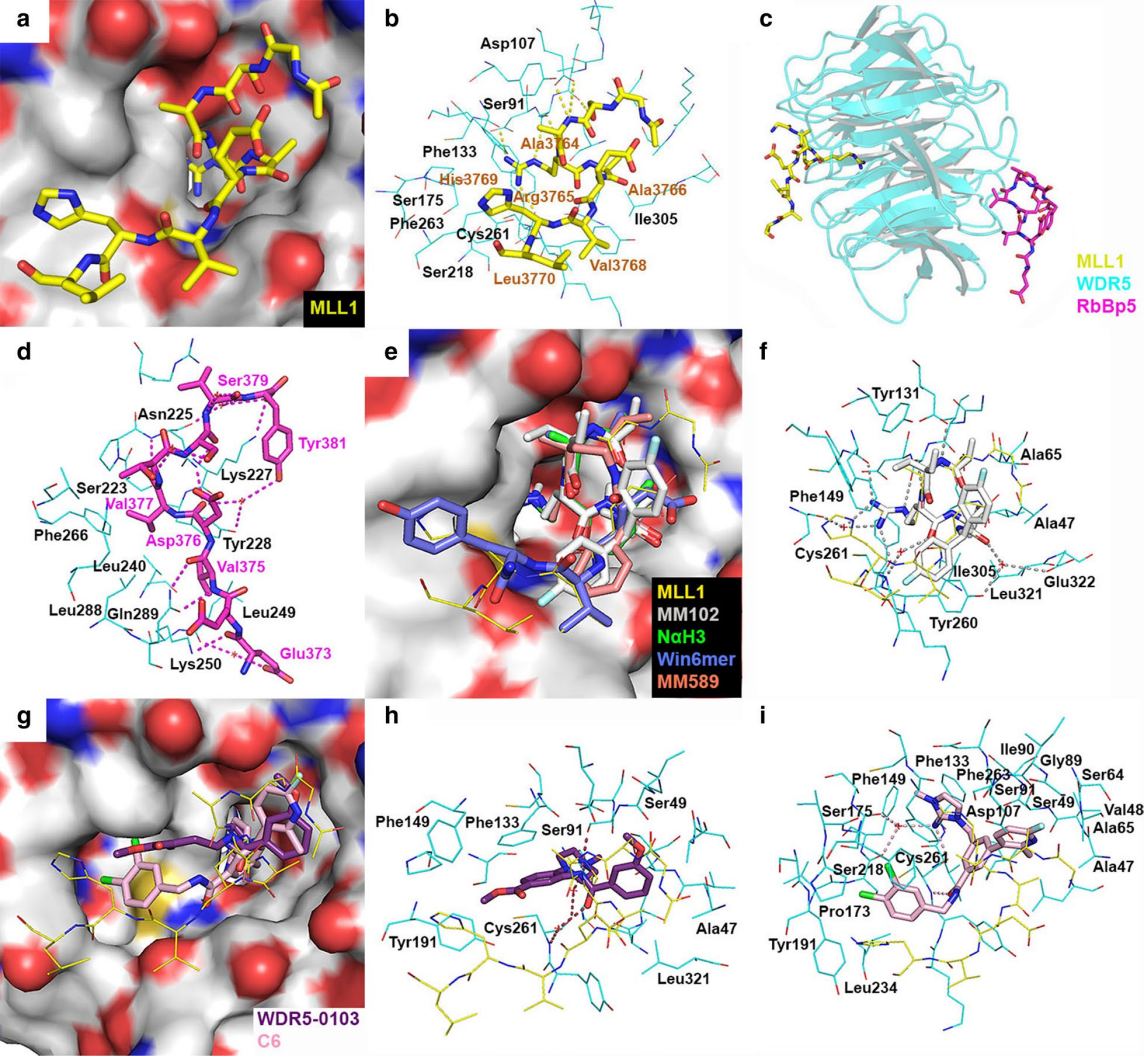
X-ray structures of the PPIs involving MLL1 SET and WDR5 in complex with its inhibitors. **a** A close-up view of the binding site of MLL1 peptide in WDR5 (PDB: 3EG6). **b** A close-up view of the WDR5-MLL1 interaction. **c** Overview of the MLL1-WDR5-RBBP5 complex (PDB: 3P4F). WDR5 is shown as cartoon in cyan and RbBP5 and MLL1 as stick models with C atoms in magentas and yellow, respectively. **d** A close-up view of the RbBP5-WDR5 interaction. **e** The active site of WDR5-MLL1 complex (PDB: 3EG6), WDR5-NαH3 (PDB: 3PSL), WDR5-Win6mer (PDB: 5SXM), WDR5-MM102 (PDB: 4GM8) and WDR5-MM589 (PDB: 5VFC). **f** The active site of WDR5-MM102 (PDB: 4GM8), superimposed with the structure of WDR5-MLL1 complex (PDB: 3EG6). **g** The active site of WDR5-MLL1 complex (PDB: 3EG6), WDR5-WDR5-0103 (PDB: 3UR4) and WDR5-C6 (PDB: 6E23). **h** The active site of WDR5-WDR5-0103 (PDB: 3UR4), superimposed with the structure of WDR5-MLL1 (PDB: 3EG6). **i** The active site of WDR5-C6 (PDB: 6E23), superimposed with the structure of WDR5-MLL1 (PDB: 3EG6). Compounds NαH3, Win6mer, MM102, MM589, WDR5-0103 and C6 are shown as stick models with C atoms in green, blue, grey, orange, purple and pink, respectively. In these structures, WDR5 is shown as an electrostatic surface and the MLL1 peptide as a line model with C atoms in yellow. Hydrogen bonds are shown as dashed lines

The crystal structure of WDR5 in complex with an MLL1 Win-derived 13- or 17-mer peptide (PDB: 3EG6, 3EMH, and 4ESG) show the peptide occupies a funnel-like pocket of WDR5 (Fig. 7a) [81–83], with the critical residue Arg3765 (of MLL1) deeply inserted into the narrow opening. Arg3765 has hydrogen bond and electrostatic interactions with Phe133, Cys261 and Ser91 of WDR5, together with hydrophobic interactions with Ser175, Ser218, Phe263, and Ile305 that surround its carbon sidechain. These interactions are essential for the MLL1-WDR5 binding, as a point mutation of Arg3765 abrogates the PPI [80]. Furthermore, the F133A mutation of WDR5 also significantly decreased the interaction between MLL1 and WDR5 in biochemical study [80, 165]. In addition, Ala3764 and Ala3766 have hydrogen bond as well as hydrophobic interactions with WDR5 residues Ser91 and Asp107. Val3768, His3769 and Leu3770 of the peptide recognize a nearby surface pocket of WDR5. The binding affinity of MLL1 to WDR5 was determined with *K*_d_ values of 0.12–1.7 μM [80, 81] (Table 1).

In addition, the ternary structure of WDR5 in complex with RbBP5 and MLL1 was determined (PDB: 3P4F), which reveals structural basis for WDR5 to bridge MLL1 and RbBP5 [79]. In this complex, the RbBP5 and MLL1 peptide bind to the opposite sides of WDR5 (Fig. 7c). MLL1 binds to WDR5 similarly as found in the MLL1-WDR5 binary complex. There are a network of hydrogen bonds stabilizing the RbBP5-WDR5 interaction (Fig. 7d). RbBP5 residues Asp376, Val377, Ser379 form multiple hydrogen bonds with WDR5 residue Asn225. Glu373, Val375, and Tyr381 of RbBP5 also interact with WDR5 residues Lys250, Gln289, and Lys227 through hydrogen bonds. In addition, Glu373 and Asp376 of RbBP5 engage in a water molecule-mediated hydrogen bond interactions with Leu249 and Tyr228 of WDR5. Tyr381 of RbBP5 also forms two water-mediated hydrogen bonds with Tyr228 and Asn225 of WDR5. Moreover, there are favorable hydrophobic interactions between RbBP5 residues Val375 and Val377 and WDR5 residues Leu249, Tyr228, Leu240, Lys250, Phe266, Ser223, and Leu288. Mutagenesis studies showed that mutation of WDR5 residues Asn225 and Gln289 resulted in > 20-fold decreased binding affinity to RbBP5 as well as ~ threefold reduced HMT activity. Similarly, mutations of RbBP5 residues Val375 and Val377 also reduces its affinity to WDR5 with a considerably decreased catalytic activity.

#### WDR5-binding compounds that inhibit MLL1 SET methyltransferase.

A histone H3-based peptide NαH3 (AcARTKQA) was developed and showed high binding affinity (*K*_d_ = 0.13 μM) to WDR5 as well as inhibited the KMT activity of MLL1 with an IC_50_ value of 4.1 μM [87]. The X-ray structure of the WDR5-NαH3 complex (PDB: 3PSL) shows that the peptide occupies the binding pocket of the MLL1 peptide and mimics its binding pose (Fig. 7e, f). An MLL1 Win-derived 12-mer peptide (GSARAEVHLRKS) was found to bind WDR5 with a *K*_d_ of 1.7 μM [81] and modestly inhibit H3K4 methylation with an IC_50_ of ~ 400 μM [80]. Subsequently, a structure-based design led to the finding of a Win-derived 6-mer peptide Win6mer (ARTEVY), which exhibited considerably increased binding affinity to WDR5 with a *K*_d_ value of 2.9 nM [88]. The X-ray structure of the WDR5**-**Win6mer complex (PDB: 5SXM) shows it exhibits a similar binding pose (Fig. 7e, f). Win6mer selectively inhibited MLL1′s KMT activity with an IC_50_ value of 2.3 μM [88].

**Fig. 8:**
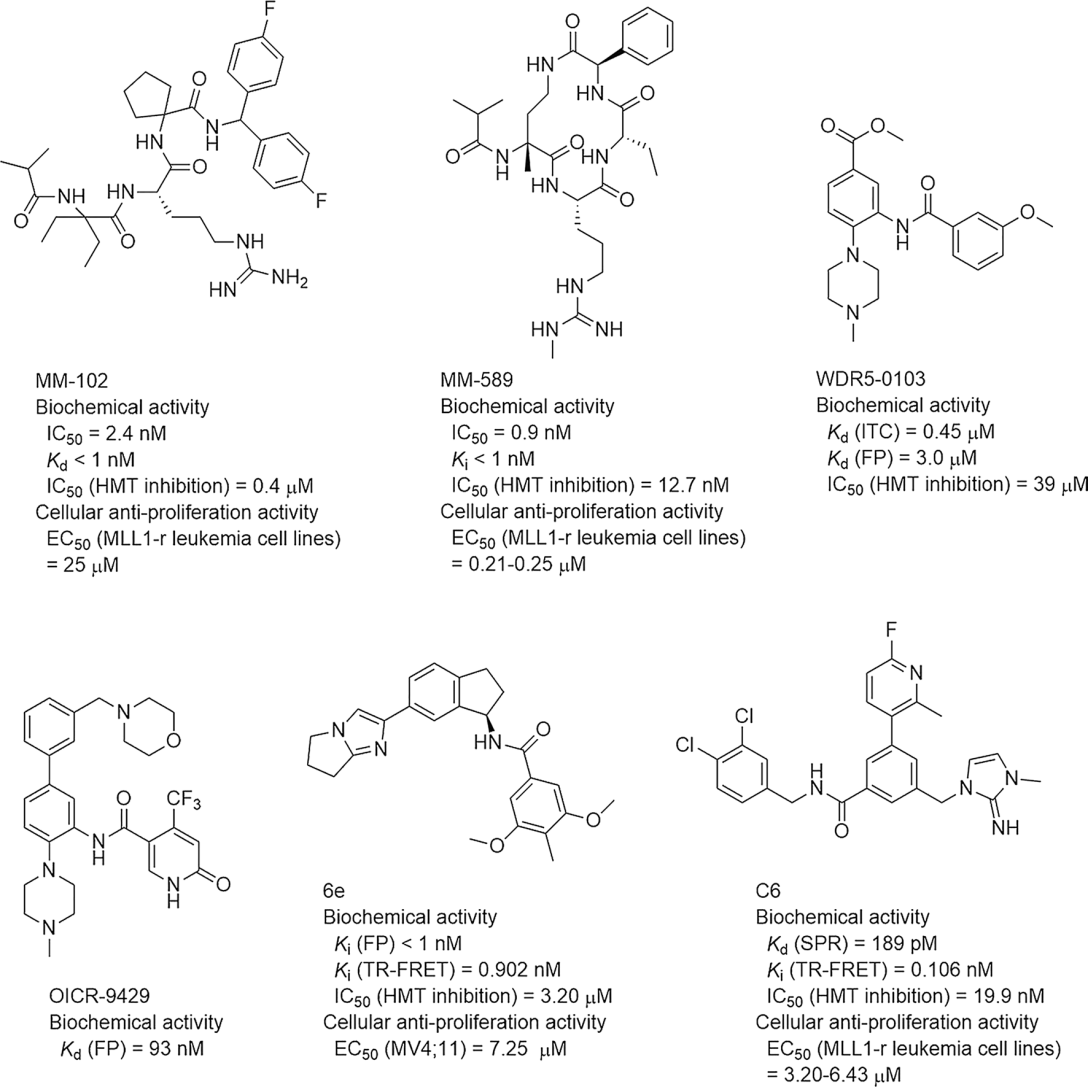
Inhibitors of the MLL1-WDR5 interaction

Efforts on shortening the 12-mer Win-derived peptide yielded a 3-mer peptide Ac-ARA-NH_2_, which showed a *K*_i_ of 120 nM [89]. Further investigation found an H3-derived 3-mer peptide Ac-ART-NH_2_ exhibited a higher binding affinity (*K*_i_ = 20 nM) [89]. Modifications based on Ac-ARA-NH_2_ produced a peptidomimetic compound MM-102 (Fig. 8) with a *K*_d_ of < 1 nM and IC_50_ of 400 nM against KMT. It also inhibited proliferation of MLL1-r leukemia cells with an EC_50_ of ~ 25 µM [84]. Its structure in WDR5 (PDB: 4GM8) shows that in addition to the interactions between the MLL1 peptide and WDR5, MM-102 has additional interactions with the protein (Fig. 7e, f). The two fluorophenyl groups of MM-102 make hydrophobic interactions with WDR5 residues Ala47, Ala65, Tyr131, Phe149, Ile305, and Leu321. The carbonyl of its Arg moiety interacts with the backbone of Cys261 of WDR5. The carbonyl close to the fluorophenyl groups forms a water-bridged hydrogen bond interaction with Tyr260 and Glu322. Macrocyclic peptides are also inhibitors [36, 90], such as 14-membered cyclic peptide MM-589. It showed more binding affinity to WDR5 as well as inhibitory activity against H3K4 methylation (IC_50_ = 12.7 nM) [90]. Crystallography studies (Fig. 7e, PDB: 5VFC) revealed that MM-589 with the small macrocyclic ring nicely fits into the funnel of WDR5. MM-589 selectively inhibited proliferation of MLL1-r leukemia cells with EC_50_s of ~ 250 nM, significantly more potent than other WDR5 inhibitors.

Through compound screening, a series of N-(2-(4-methylpiperazin-1-yl)-5-nitrophenyl)benzamide compounds have been discovered to be small-molecule inhibitors of the MLL1-WDR5 interaction, with compound WDR5-0103 (Fig. 8) being the most potent [91]. It exhibited a low micromolar binding affinity to WDR5 in biophysical assays and selectively inhibited the KMT activity with an IC_50_ of 39 µM. Its crystal structure in WDR5 (PDB: 3UR4) shows its binding is quite different from that of the MLL1 peptide (Fig. 7g, h). The positively charged *N*-methylpiperazine group (at the physiological *p*H) mimics Arg of the peptidic inhibitors and is inserted into the WDR5 deep tunnel (Fig. 7g, h), with its distal N atom forming a water-mediated hydrogen bond with Cys261. The central phenyl ring has a π-π interaction with Phe133, with its attached ester moiety well fitted in a hydrophobic cleft formed by Tyr191 and Phe149. The amide group interacts with Cys261 through a water-bridged hydrogen bond. In addition, the methoxylphenyl moiety is located a shallow cavity surrounded by residues Ala47, Ser49, Ser91, and Leu32. Modifications based on compound WDR5-0103 furnished inhibitors with considerably improved affinity [85, 86, 92–96], such as OICR-9429 (*K*_d_ = 93 nM, Fig. 8) [85, 86]. In cell-based assays, OICR-9429 inhibited proliferation and induced the differentiation of AML patient cells.

An NMR-based fragment screening has identified a series of imidazole-containing inhibitors, with compound 6e (Fig. 8) being the most potent with a *K*_i_ value of < 1 nM. This compound moderately inhibited the KMT activity (IC_50_ = 3.2 µM) [97]. It also exhibited a modest anti-proliferative activity against MLL1-r leukemia cells. The same strategy led to the discovery of compound C6 (Fig. 8) with a *K*_d_ = 0.2 nM (to WDR5) as well as IC_50_ of 20 nM against H3K4 methylation [98–100]. The X-ray structure of the WDR5-C6 complex (PDB: 6E23) shows that its basic 2-imino-2,3-dihydroimidazole group mimics the Arg of the MLL1 Win peptide (Fig. 7g, i). It forms direct and water-bridged hydrogen bonds and has electrostatic interactions with Ser175, Ser218 and Cys261. Moreover, the imidazole ring is sandwiched in between the aromatic side chains of Phe133 and Phe263 with favorable π-π interactions. The amide group of compound C6 forms a hydrogen bond with Cys261. The lipophilic dichlorobenzyl group resides in a hydrophobic pocket formed by the side chains of Phe149, Pro173, Tyr191, Leu234, Ser218 and Cys261. In addition, the binding of C6 is further enhanced by the hydrophobic interactions of its pyridine moiety with a hydrophobic pocket surrounded by Ser91, Asp107, Ile90, Gly89, Ser49, Ser64, Ala65, Val48, and Ala47. In cell-based assays, compound C6 was found to selectively inhibit the proliferation of MLL1-r leukemia cells with EC_50_ values of 3–6 µM. Moreover, piribedil [101], rabeprazole [102], DC_M4_1 and DC_M5_2 [103] were reported to be small-molecule inhibitors of the MLL1-WDR5 interaction.

### Other PPIs involving MLL1

Several other PPIs involving MLL1 play important roles in health and diseases. For example, the MLL-N and MLL-C interaction through their FYRN and FYRC domains is important for the stability and functions of MLL1 [42] and disruption of such interaction by expression of a competing FYRC peptide caused significant MLL1 degradation [166]. In addition to its DNA-binding ability, MLL1′s CxxC domain interacts with PAFc, which facilitates MLL1 binding to its target genes (e.g., HoxA9 and Meis1) for transcription [15, 29]. Inhibition of the MLL1-PAFc interaction by expression of a competing MLL1 peptide blocked MLL1-mediated gene expression as well as onco-MLL1-induced leukemogenesis [167]. In addition to CBP/p300 described above, MLL1′s TAD domain interact with other histone/protein acetyltransferases MOZ and MOF for target gene expression activation [32, 160]. However, these PPIs have not been well characterized and their biochemistry, structural biology and pharmacological inhibition await further investigation.

## PPIs involving MLL1 fusion proteins

In MLL1-r leukemia, chromosome translocations produce an oncogenic fusion protein consisting of the N-terminal DNA-interacting domains of MLL1 (~ 1400 residues) fused with one of > 70 fusion partner proteins (Fig. 1a) [12–14], among which transcription cofactors AF9 and its paralog ENL, AF4 (also known as AFF1) and its paralog AFF4, and ELL are found in > 70% MLL1-r leukemias (Fig. 1b) [10, 38]. These proteins have been found to associate with each other in several isolated transcription complexes, which have been commonly named super elongation complexes (SEC) [25, 39, 40]. Formation of SEC is required for expression of characteristic genes of MLL1-r leukemia (e.g., HoxA9 and Meis1) as well as leukemia transformation. Moreover, SEC was also found to be recruited by MLL1-AF6 and -AF10, two other common fusion oncogenes (Fig. 1b) [25, 40]. The common feature of these frequent MLL1 fusion partners is their ability to recruit SEC and other associated proteins.

**Fig. 9:**
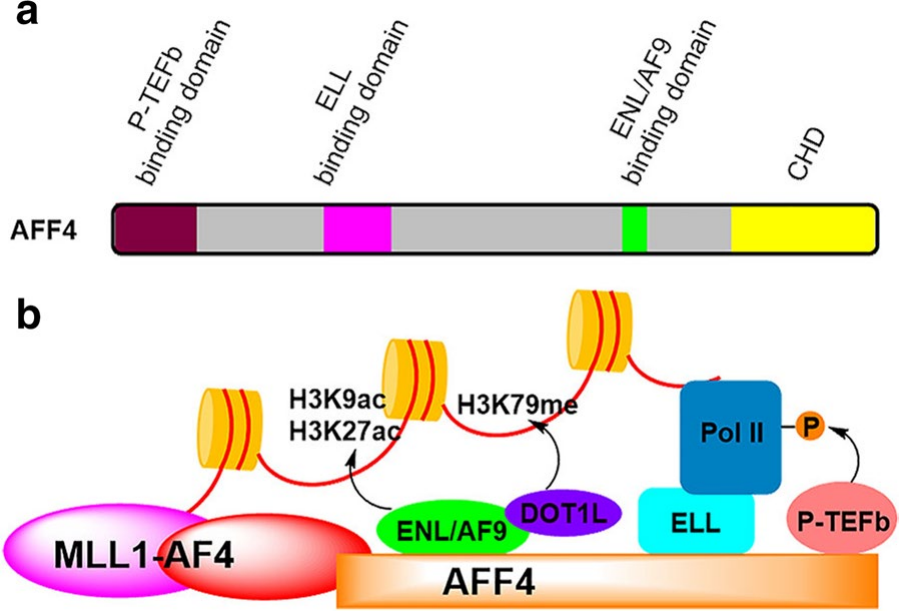
**a** Illustration of AFF4, showing functional domains of P-TEFb binding domain (residues 1–73), ELL binding domain (301–350), ENL/AF9 binding domain (761–774), and the CHD domain (899–1163). **b** A schematic illustration showing how MLL1-AF4 oncoprotein recruits SEC as well as the biological functions of these proteins for gene regulation

Together with P-TEFb (positive transcription **e**longation **f**actor), MLL1 fusion partners AF4/AFF4, AF9/ENL and ELL are essential members of SEC. AF4 and its paralog AFF4 are the scaffold for the formation of SEC. AF4/AFF4 contain intrinsically disordered binding domains for P-TEFb, ELL, and AF9/ENL as well as a structured C-terminal homology domain (CHD) (Fig. 9a) [168]. Figure 9b schematically illustrates the recruitment of SEC by MLL1-AF4. Other major onco-MLL1 (e.g., MLL1-AF9/ENL and -ELL) can recruit SEC similarly. MLL1(1–1400) functions as a transcription factor to bind to its target gene promoters. AF4/AFF4 form a heterodimer, which interact with other SEC member proteins and serve as a scaffold for SEC assembly. P-TEFb, consisting of cyclin-dependent kinase 9 (CDK9) in complex with regulatory cyclin-T1 protein, phosphorylates Pol II, while ELL binds Pol II and enhances its activity [169, 170]. Both of these two events are key steps for Pol II to leave promoter-proximal pausing and start transcription elongation of a gene. AF9 and homologous ENL contain an N-terminal YEATS (Yaf9, ENL, AF9, Taf14, and Sas5) and a C-terminal AHD (ANC1 homology domain) domain. The conserved YEATS domain recognizes and binds acetylated H3K9 or H3K27 [171, 172]. The AHD domain of AF9/ENL plays critical roles in MLL-r leukemia. AHD is found in all fusion MLL1-AF9/-ENL, while YEATS is lost in almost all clinical variances of MLL-AF9/-ENL [25]. Transformation of murine hematopoietic progenitor cells with MLL1 fused with various truncated forms of AF9 confirmed that AHD is essential for MLL-AF9/ENL leukemia, but YEATS is dispensable [25, 173]. The AHD domain of AF9/ENL interacts with AF4/AFF4, which is critical to the formation of SEC as well as MLL1-AF9/ENL-mediated SEC recruitment. Moreover, AF9/ENL AHD’s another binding partner DOT1L is a H3K79 methyltransferase [174, 175], which can methylate H3K79 (Fig. 9b). Hypermethylation of H3K79 has been found to be characteristic to MLL1-r leukemias [176]. DOT1L has been found to be required for expression of MLL1-target genes such as HoxA9 and Meis1 and therefore a drug target for MLL1-r leukemia. During the past few years, potent inhibitors of DOT1L have been developed [177–184], showing potent and selective activities against MLL1-r leukemia. An advanced DOT1L inhibitor EPZ-5676 [184] has been in clinical trials against the malignancy.

In addition to MLL1-r leukemia, SEC has been found to play critical roles in transcription elongation of HIV gene in the human genome [185–188]. Pol II is recruited to the HIV promoter and initiated transcription of HIV gene, but after ~ 50 transcripts, transcription is paused in a transactivating response region (TAR). HIV viral protein Tat is a transactivator protein, which binds to TAR and recruits SEC. This event causes phosphorylation of Pol II and release from transcription pausing. Moreover, SEC also regulates expression of transcription factor Myc, a master regulator for cancer cell growth, implicating such PPIs’ roles in other cancers and HIV infection. Biological functions of SEC were recently reviewed [189]. Table 3 summarizes the binding affinity, availability of the X-ray or NMR structure and inhibitors of the PPIs involving MLL1 fusion partners.

**Table 3:** Binding affinity (*K*_d_), structure, and inhibitors of the critical PPIs involving MLL1 fusion proteins

PPIs	*K*_d_ (μM)	Structures (PDB)	Inhibitors
AFF4-ELL2	0.086 [190]	5JW9 [190]	None
AFF4-Cyclin T1	0.1 [191]	4IMY [191]	[192]
AFF4-P-TEFb	0.01 [191]	4IMY [191]	[192]
AFF4-Tat-P-TEFb	0.0009 [191]	4OR5 [193]	None
AF9(AHD)-AF4	0.0002 [194]	2LM0 [194]	[195]
AF9(AHD)-DOT1L	0.0016 [194]	2MV7 [196]	[197]
ENL(AHD)-DOT1L	0.2 or 1.34 [198]	None	None
AF9 or ENL AHD-CBX8	> 0.5 or 0.11 [199]	2N4Q [199]	None
AF9 or ENL AHD-BCoR	0.018 or 0.002 nM [199]	6B7G [199]	None
AF9(YEATS)-H3K9ac	3.7 [172]	4TMP [172]	[200–204]
ENL(YEATS)-H3K9ac	32.2 [205]	None	[200–204]
ENL(YEATS)-H3K27ac	30.5 [205]	5J9S [205]	[200–204]

### AF4-AFF4 heterodimerization

#### Biological function

AF4 and AFF4 share a high degree of homology[206], particularly for their domains involved in PPIs. AFF4 is required for MLL1-r leukemia, as its knockdown resulted in the decreased expression of MLL1 target genes [39]. Formation of AF4-AFF4 heterodimer has been found to be preferred biochemically [168] and in cells [25] over their homodimers. Further investigation using MLL1-AF4 and AFF4 with deletion of various AF4/AFF4 domains showed that only the CHD domain of AFF4/AF4 is required for leukemic transformation, while other AF4/AFF4′s interacting domains with P-TEFb, ELL and AF9/ENL are dispensable for MLL1-AF4/AFF4 leukemia [39]. These results underscore the critical role for CHD-mediated AF4-AFF4 heterodimerization in the leukemia and it is therefore a potential drug target for MLL1-r leukemia. However, there have been no inhibitors of the PPI.

#### Structure

**Fig. 10:**
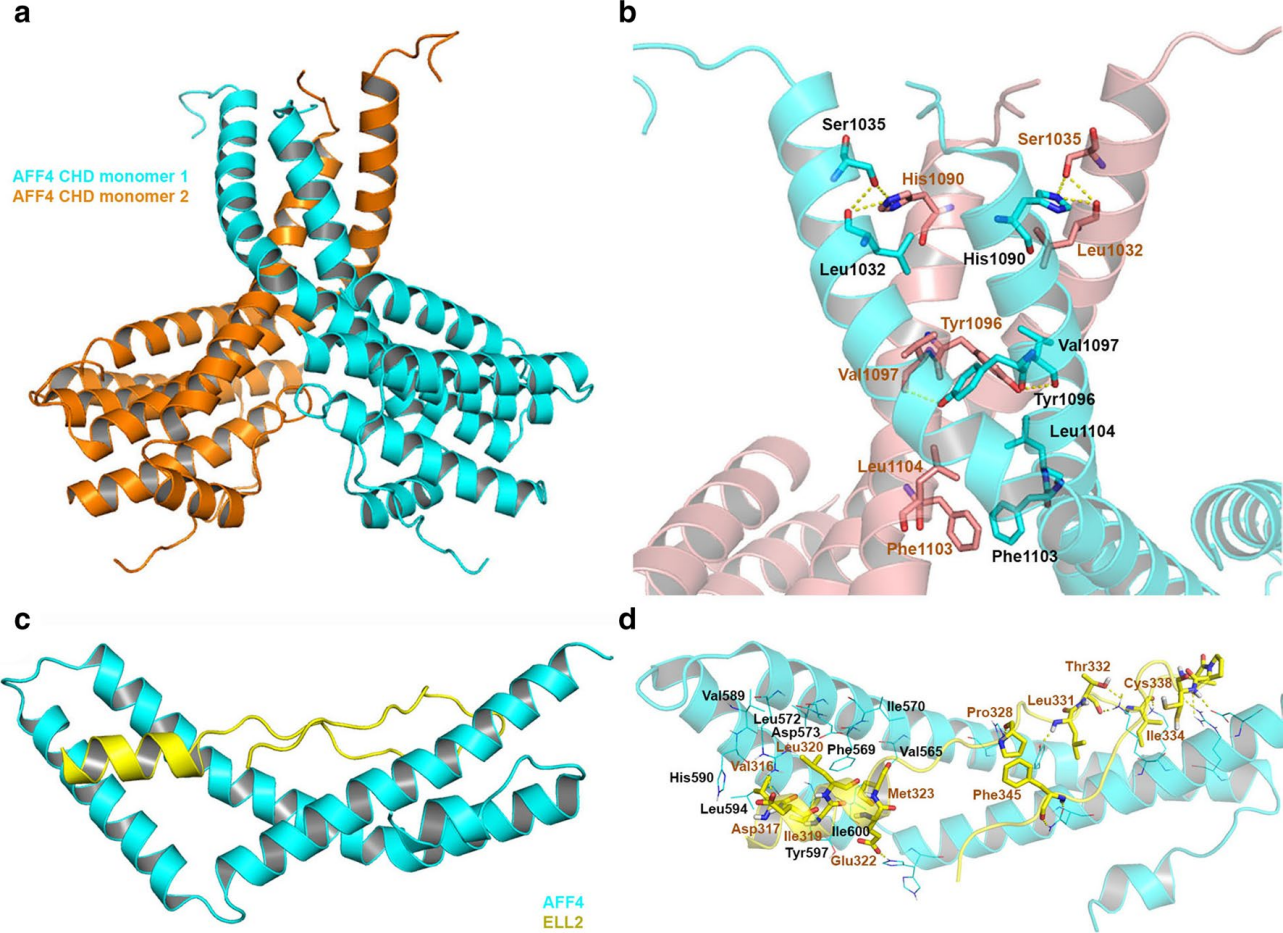
X-ray structure of AFF4 CHD homodimer (PDB: 6R80) and AFF4 in complex with ELL2 (PDB: 5JW9). **a** Overview of AFF4 CHD homodimer. **b** A close-up view of the dimer interface. The AFF4 CHD homodimer is presented as cartoon models with one monomer in cyan and the other in orange. **c** Overview of the AFF4-ELL2 complex. **d** A close-up view of the AFF4-ELL2 interaction. The AFF4-ELL2 complex is presented as cartoon models with AFF4 in yellow and ELL2 in cyan. Hydrogen bonds are shown as yellow dashed lines

Although the crystal structure of AF4-AFF4 heterodimer is not available, that of the homodimer of AFF4 CHD domain has been determined (PDB: 6R80) [168, 207, 208]. Biochemical including mutagenesis studies revealed that the interface between AF4 and AFF4 heterodimer overlap with that of AFF4 homodimer, thanks to a high degree of homology between the two CHD domains. Each monomer of AFF4 CHD contains 8 α-helices, among which the two longest helices together with the loop between them participate in homodimerization (Fig. 10). They form a large hydrophobic surface, mainly consisting of His1090, Tyr1096, Val1097, Phe1103, and Leu1104, interacting with that of the other monomer. Replacement of these key residues with alanine disrupted AFF4 dimerization in solution. In addition to the hydrophobic interactions, a network of hydrogen bond interactions among Leu1032, Ser1035, His1090, Tyr1096, and Val1097 contribute to the stability of the homodimer (Fig. 10b).

### AF4/AFF4-ELL interaction

#### Biological function

ELL (also known as ELL1) is also a major MLL1 fusion partner (Fig. 1b). ELL as well as its paralog ELL2 was discovered as a RNA polymerase elongation factor [209], which has been found to be a core component of SEC and required for transcription elongation for a number of genes [39]. Biologically, ELL binds to Pol II and increases the catalytic activity of the Pol II elongation complex [169, 170], facilitating transcription elongation of a gene. Similar to MLL1-AF4, MLL1-ELL can recruit the other member proteins through the ELL-AF4/AFF4 interaction to form SEC for leukemic transformation. The AF4/AFF4-ELL interaction is a potential drug target for MLL1-ELL and/or other MLL1-r leukemia. No inhibitors have been found for the PPIs between AF4/AFF4 and ELL1/2.

#### Structure

X-ray structure of a fusion protein consisting of AFF4(318–337) and ELL2 C-terminal domain (519–640) was determined (PDB: 5JW9) [190], providing the structure basis for the PPI. ELL2 forms 4 α-helices and folds into an arch-shaped conformation, with its large concave surface to interact with AFF4. The AFF4 peptide contains a small α-helix followed by an extended hairpin. The α-helix of AFF4 occupies a hydrophobic groove constituted by the first two α-helixes of ELL2 at the N-terminal (Fig. 10c, d). The AFF4 residues Val316, Ile319, Leu320 and Met323 have mostly hydrophobic interactions with the residues Val565, Phe569, Ile570, Leu572, Asp573, Val589, His590, Tyr597, Leu594, and Ile600 of ELL2. In addition, there are extended hydrophobic interactions between the AFF4 hairpin and ELL2 hydrophobic surface. Moreover, a network of hydrogen bond and electrostatic interactions between the AFF4 residues Asp317, Glu322, Pro328, Leu331, Thr332, Ile334, Cys338 and Phe345 and ELL2 also contribute to the binding, giving a strong binding affinity with *K*_d_ values of 0.086–4.0 μM depending on assay methods (Table 3) [190]. Mutagenesis studies showed that the hydrophobic interactions between the residues of the AFF4 α-helix and ELL2 contribute the most to the binding.

### AFF4-P-TEFb interaction

#### Biological function

P-TEFb consists of a catalytic component CDK9 and an associated regulatory component cyclin-T1, playing a critical role in transcription elongation. P-TEFb is recruited to a gene promoter through the cyclin-T1-AFF4/AF4 interaction, with CDK9 phosphorylating the Serine-2 residue of Pol II and triggering transcription elongation process [210]. Onco-MLL1 recruits SEC for transcription elongation, leading to aberrant gene expression and leukemogenesis. SEC also mediates expression of the HIV gene in human genome [211, 212]. HIV protein Tat forms a ternary complex with AFF4 and cyclin-T1 [193] and thus recruits SEC to HIV gene promoter for transcription elongation. Pharmacological inhibition of CDK9 was found to block Pol II-mediated gene transcription [213] as well as HIV gene transcription and viral replication [214]. Therefore, targeting the PPIs between cyclin-T1 and AFF4/AF4 represents a potential therapeutic approach to the treatment of MLL1-r leukemia and HIV infection. Moreover, targeting the PPI may only affect SEC-regulated gene transcription and spare recruitment of P-TEFb by other transcription cofactors. Given the critical roles of P-TEFb in aberrant gene expression in MLL-r leukemia, targeting the cyclin-T1-AFF4 interaction is selective and could be less toxic.

#### Structure

**Fig. 11:**
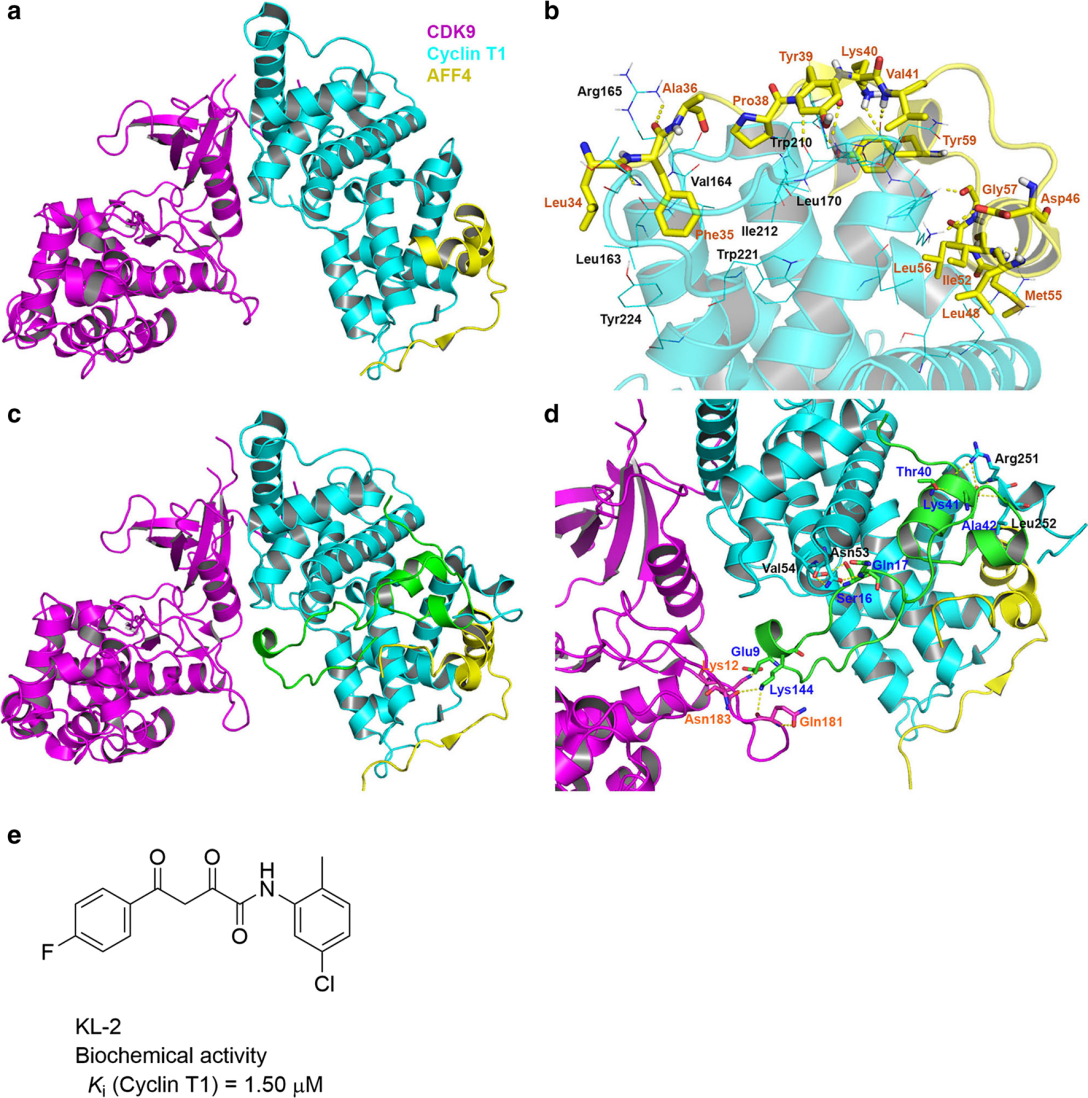
X-ray structures of P-TEFb in complex with AFF4 and the structure of SEC disruptor. **a** Overview of P-TEFb-AFF4 (PDB: 4IMY). **b** A close-up view of the AFF4-P-TEFb(Cyclin T1) interactions. **c** Overview of the ternary P-TEFb-Tat-AFF4 complex (PDB: 4OR5). **d** A close-up view of the Tat-P-TEFb-AFF4 interface. AFF4 is shown in yellow, CDK9 in magenta, Cyclin T1 in cyan, and Tat in green. Hydrogen bonds are shown as yellow dashed lines. **e** Structure of SEC disruptor

The crystal structure of P-TEFb in complex with an AFF4 peptide (PDB: 4IMY) [191] shows AFF4 binds to Cyclin-T1 through a combination of hydrophobic, hydrogen-bond and other polar interactions (Fig. 11a, b), while it does not interact with CDK9. Residues Leu48, Ile52, Met55, Leu56 and Tyr59 in the two α-helices of AFF4 sit in a shallow groove of Cyclin-T1 with hydrophobic interactions. Cyclin T1′s Leu163, Val164, Arg165, Trp221, and Tyr224 residues form a hydrophobic cleft, which accommodates the AFF4 residues Leu34, Phe35 and Ala36. Another hydrophobic pocket of Cyclin-T1 (Trp210, Leu170 and Ile 212) holds the Pro38 residue of AFF4. In addition, Leu34, Phe35, Tyr39, Lys40, Val41, Asp46, Met55, Gly57, and Tyr59 of AFF4 form multiple hydrogen bonds and have electrostatic interactions with Cyclin T1. While the binding affinity between AFF4 and Cyclin T1 alone is high (*K*_d_ = 102–130 nM), formation of P-TEFb significantly increases the binding affinity with *K*_d_ values of 10–36 nM [191] (Table 3).

X-ray structures of the quaternary Tat-AFF4-P-TEFb complexes were determined (PDB: 4OR5) [191, 193]. Cyclin-T1 of P-TEFb served as a receptor platform for the binding of the Tat and AFF4 peptides (Fig. 11c, d). CDK9 resides in the other side of Cyclin-T1 without interactions with AFF4, while the remote residues Glu9 and Lys12 of Tat can reach CDK9 and form multiple hydrogen bond interactions with the residues Lys144, Asn183 and Gln181 of CDK9. The binding of Tat to Cyclin T1 relies on multiple hydrophobic, hydrogen bond, and electrostatic interactions. Hydrogen bonds and other polar interactions between Ser16 and Gln17 of Tat and Val54 and Asn53 of Cyclin T1 constitute an important anchor point, while another major anchoring site is located between the Tat residues Thr40, Lys41, and Ala42 and Cyclin T1′s Arg251 and Leu252. Furthermore, participation of Tat significantly (> tenfold) enhances the binding affinity of between AFF4 and P-TEFb complex [191] (Table 3).

#### Inhibitors

Compound KL-2 (Fig. 11e) was reported to bind to Cyclin T1 at a low micromolar concentration and inhibit the Cyclin T1-AFF4 interaction [192]. The disruption of the PPI resulted in reduced SEC-dependent transcriptional responses and downregulation of Myc expression as well as Myc-dependent transcriptional programs. KL-2 can also suppress tumor growth in vitro and in a mouse model of Myc-dependent cancer. However, it is noted that KL-2 containing an electrophilic, chemically reactive α,γ-di-keto-amide group could covalently bind to a protein non-specifically. Specificity as well as possible off-target effects of this compound might need to be examined carefully.

### PPIs involving the AHD domain of AF9 and its paralog ENL

#### Biological function

AF9 and its paralog ENL contain an N-terminal YEATS domain (~ 110 residues) and a C-terminal AHD domain (~ 70 residues). AF9 [215] and ENL [9] were first identified as a fusion partner of MLL1. Later they were found to be homologous nuclear proteins that play a role in activating gene transcription in lymphoid and myeloid cells, with their AHD domain required for the activity [216]. ENL is required for MLL1-r leukemia [25] as well as a broader range of AMLs [205], as its knockdown inhibited aberrant gene expression and cell proliferation of these cells.

The AHD domain alone is intrinsically disordered, but it binds a short peptide segment of DOT1L or AF4/AFF4 with a consensus sequence of LxVxIxLxxV/L and forms a structured complex [194, 196]. High affinity binding between AF9 and DOT1L or AF4 (Table 3) is required for oncogenesis of MLL-AF9. MLL1-AF9 with D546R or D544R mutation, which has a significantly reduced affinity to DOT1L or AF4, failed to transform murine hematopoietic stem/progenitor cells [173]. Disruption of AF9-AF4 interaction by an AF4-derived peptide inhibited the cell proliferation with enhanced cell apoptosis [217, 218], while it did not affect the proliferative of normal hematopoietic cells. These results suggest inhibiting the AF9/ENL-AF4/AFF4/DOT1L interactions could be useful to treat MLL1-r leukemia.

In addition to AF4/AFF4 and DOT1L, AF9/ENL AHD interacts with CBX8 (**c**hromo**b**o**x** homolog **8**) [219, 220] and BCoR (**B**CL-6 **cor**epressor) [221] with a similar binding mode [194]. BCoR also possesses the consensus sequence of LxVxIxLxxL and exhibits a high-affinity binding to AF9 AHD (*K*_d_ = 32 nM), while CBX8 with LxAxIxLxxI has 30 × less affinity [194]. Binding of AF9/ENL with their binding partners is mutually exclusive. The interactions between AF9/ENL and CBX8 or BCoR were also found to be critical for MLL1-AF9/-ENL induced leukemic transformation through gene knockdown and other biological experiments in vitro and in vivo [219, 220, 222, 223].

The YEATS domain of AF9/ENL is conserved from yeast to humans, which was found to recognize and bind acetylated H3K9 and H3K27 [172, 205]. Binding to H3K9ac facilitates AF9 to occupy its target genes and recruit DOT1L for H3K79 methylation. H3K9ac-binding deficient mutants of AF9 (F59A and Y78A) conferred the impaired localization of AF9 as well as the decreased levels of H3K79me3. Knockout of ENL significantly suppressed proliferation of a broader range of AML (including MLL1-r) cells [205]. These ENL-depleted leukemia cells can be rescued by transfecting WT ENL, but not F59A mutant ENL that cannot bind H3K27ac, showing that YEATS as well as its binding to H3K27ac is of importance. In addition, ENL knockout did not affect the growth of several solid tumors, showing ENL is only essential for leukemias. Chromatin immunoprecipitation followed by sequencing showed ENL is enriched and colocalized with H3K27ac (and H3K9ac) on the promoters of leukemia relevant genes. Moreover, disruption of the interactions between YEATS and H3K27ac through structure-based mutagenesis reduced the recruitment of Pol II to the gene promoters, causing suppression of gene expression as well as growth arrest. Another research also revealed critical roles of ENL and its YEATS-H3K27ac interaction in leukemogenesis [224].

#### Structure and inhibition of AF9/ENL-AF4/DOT1L/CBX8/BCoR

**Fig. 12:**
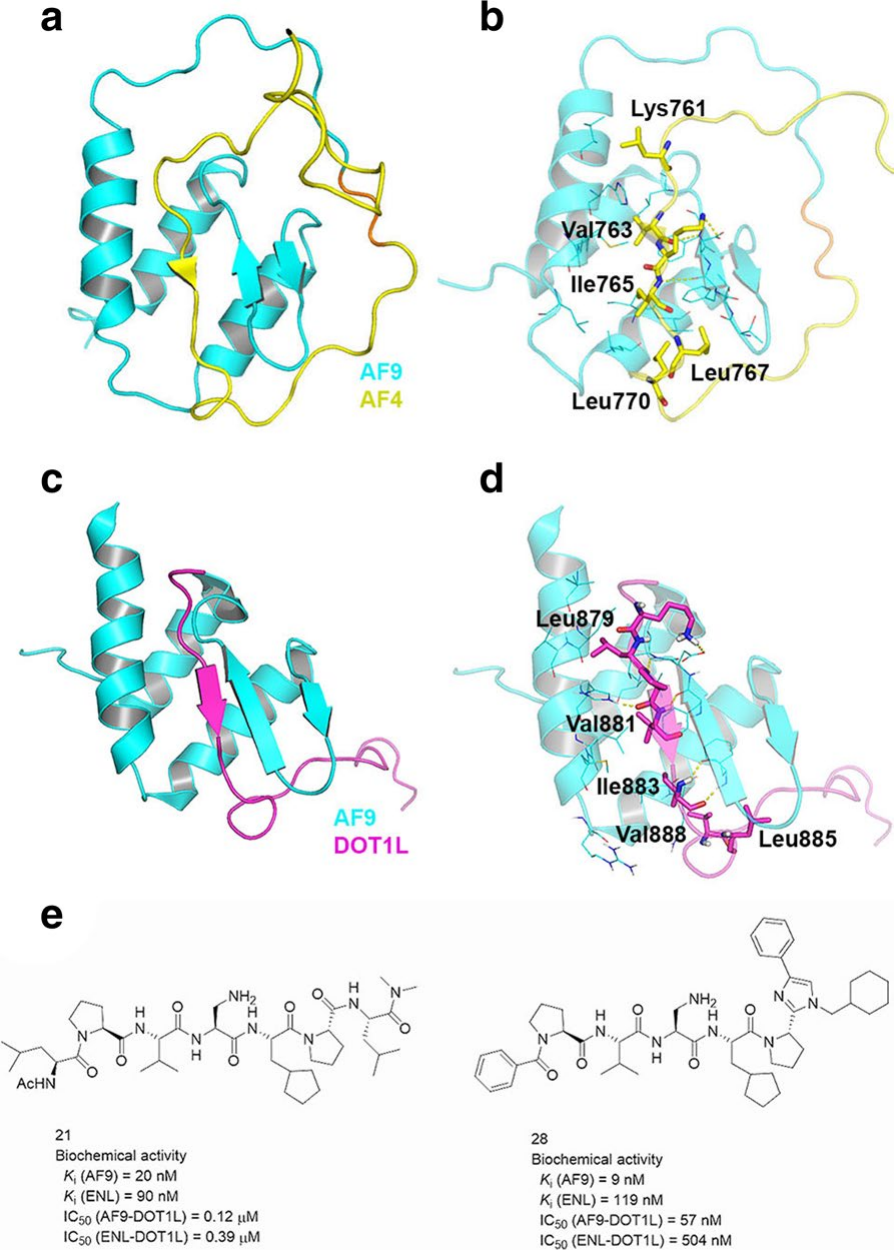
Solution NMR structures of AF9 in complex with AF4/DOT1L (PDB: 2LM0/2MV7) and inhibitors of the PPIs involving AF9/ENL. **a**, **c** Overview of AF9-AF4/DOT1L interaction. **b**, **d** A close-up view of AF9-AF4/DOT1L interaction. In these structures, AF9-AF4/DOT1L complexes are presented as cartoon with AF9 in cyan, AF4 in yellow, DOT1L in magenta and the linker (in AF9-AF4 complex) between them in orange. The AF9 residues involved in the interaction are shown as line model with C atoms in cyan and AF4/DOT1L residues shown as stick models with C atoms in yellow. The hydrogen bonds are shown as yellow dashed lines. **e** Inhibitors of the PPIs involving AF9/ENL

The NMR solution structures of fusion proteins AF9-AF4 (PDB: 2LM0) and AF9-DOT1L (PDB: 2MV7) have been determined (Fig. 12) [194, 196]. The fusion protein consists of the AHD domain of AF9 and the AF4 peptide (residues 738–779) or the DOT1L peptide (residues 3877–3900), interconnected with a flexible linker peptide. The two structures are similar. The β-strand of AF4 or DOT1L peptide is bound to a hydrophobic cleft formed by three α-helices and one β-hairpin of AF9, driven by mostly hydrophobic interactions. The consensus residues Leu761, Val763, Ile765, Leu767, Leu770 of AF4 peptide (or Leu879, Val881, Ile883, Leu885, and Val888 of DOT1L) have favorable interactions with the hydrophobic groove of AF9 AHD. Moreover, there are hydrogen bond and electrostatic interactions between AF9 and the ligand peptide to stabilize the PPIs. The solution NMR structures of AF9-CBX8 and BCoR complexes have also been solved (PDB: 2N4Q, 6B7G), in which CBX8/BCoR bind to the AF9 hydrophobic groove similarly to AF4/DOT1L [222].

AF4-derived PFWT 13-mer peptide is an inhibitor the AF9-AF4 interaction and can disrupt such PPI at the concentrations of 10–10,000 ng/mL [217, 218]. When conjugated with a penetration transporter or a penetrating peptide, these peptides can inhibit proliferation of MLL1-r leukemia cell lines, while showing no apparent toxicity to hematopoietic progenitor cells. The SPK111 peptide, which was derived from the PFWT peptide by conjugation with a modified HIV Tat protein domain, exhibited better in vivo stability and prolonged the survival of mice bearing MLL1-AF9 and MLL1-ENL rearranged leukemia cells [195].

A DOT1L 10-mer peptide was found to inhibit the AF9- and ENL-DOT1L interactions with IC_50_ values of 490 nM and 1340 nM, respectively [198]. It can also inhibit colony-forming ability of MLL1-AF9 transformed leukemic cells. Medicinal chemistry optimization yielded a series of 7-mer peptidomimetic compounds **21** and **28** (Fig. 12e) against the interaction between AF9/ENL and DOT1L with IC_50_ values as low as 57 nM [197]

### Structure and inhibition of the AF9/ENL(YEATS)-H3K27ac interaction

**Fig. 13:**
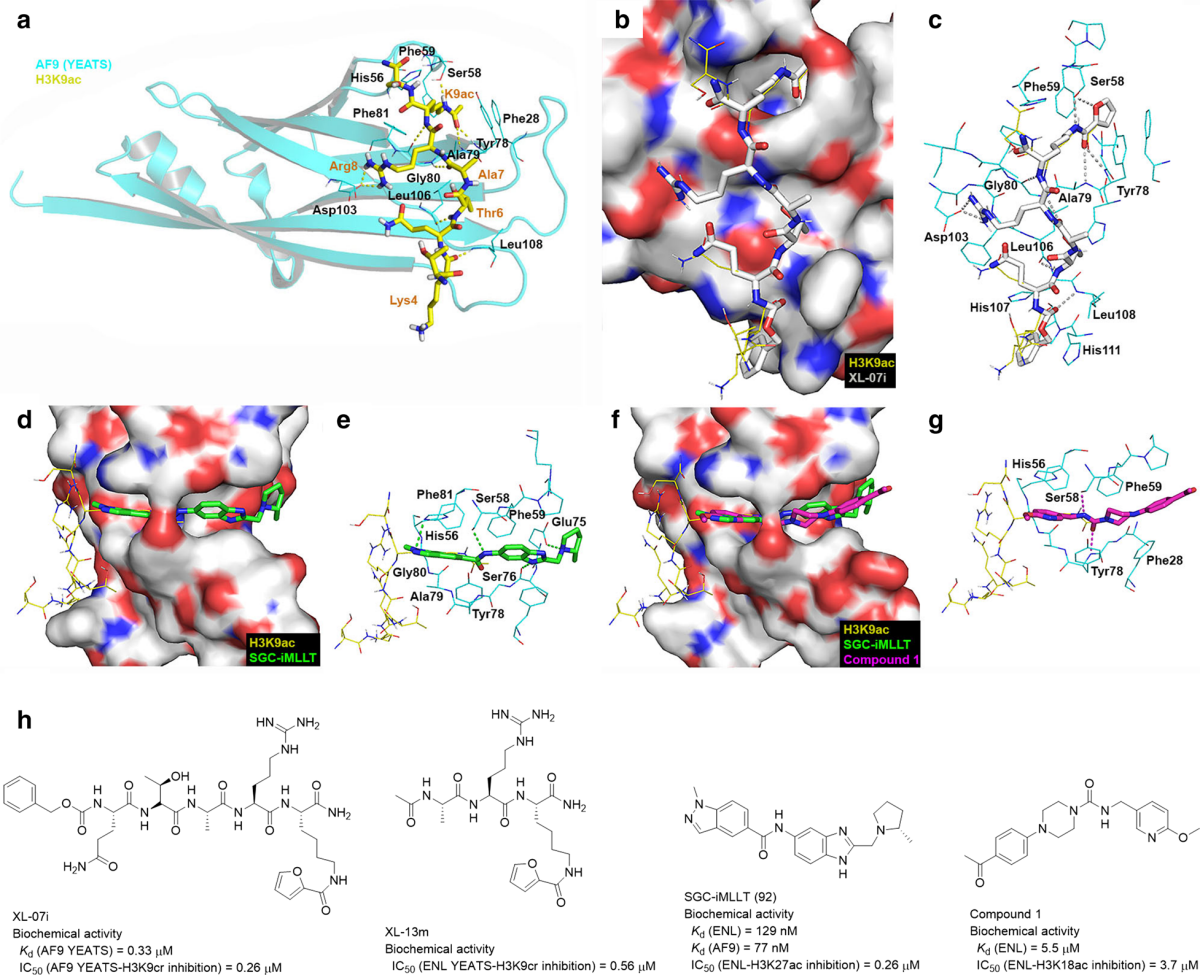
X-ray structure of AF9/ENL YEATS in complex with inhibitors and the structure of YEATS inhibitors. **a** AF9 YEATS in complex with H3K9ac (PDB: 4TMP). In this structure, AF9(YEATS) is shown as cartoon with C atoms in cyan and H3K9ac as a stick model with C atoms in yellow. **b** The active site of AF9(YEATS)-XL-07i (PDB: 5YYF), superimposed with the structure of AF9(YEATS)-H3K9ac complex (PDB: 4TMP). **c** A close-up view of AF9(YEATS)-XL-07i interaction. XL-07i is shown as a stick model with C atoms in yellow. **d**, **e** The active site of ENL-SGC-iMLLT (PDB: 6HT1), superimposed with the structure of AF9(YEATS)-H3K9ac complex (PDB: 4TMP). **f**, **g** The active site of ENL-compound 1 (PDB: 6T1I) and ENL-SGC-iMLLT (PDB: 6HT1), superimposed with the structure of AF9(YEATS)-H3K9ac complex (PDB: 4TMP). SGC-iMLLT and compound 1 are shown as stick model with C atoms in green and magenta, respectively. In structures b, d and f, AF9/ENL YEATS is shown as an electrostatic surface and H3K9ac as a line model with C atoms in yellow. Hydrogen bonds are shown as dashed lines. **h** Structures of YEATS inhibitors

X-ray structures of the YEATS domain of AF9 and ENL in complex with acetylated or crotonylated histone peptide were determined (PDB: 4TMP, 5J9S, 5HJB, 5HJD) [172, 205, 225] and these structures are similar because of the high homology between AF9 and ENL. The YEATS domain contains eight antiparallel β-strands and two α-helices (Fig. 13), which is different from BRD (which also binds an acetylated lysine) with four conserved α helices [226]. The central deep hydrophobic pocket of YEATS is responsible for recognizing of the acetylated lysine. H3K9ac is bound to YEATS in an orientation perpendicular to the β-stands with K9ac docked into an aromatic cage formed by AF9 residues Phe28, His56, Ser58, Phe59, Try78, and Phe81 with favorable hydrophobic interactions as well as hydrogen bonds with Ser58, Try78 and Ala79. Mutation of these residues resulted in significantly reduced binding affinity. In addition, acylation of K9 is important as it neutralizes the positive charge of the lysine sidechain and gains hydrophobic interactions with the pocket. In addition to K9ac, the other residues of H3K9ac peptide participate in the binding to YEATS. Lys4, Thr6, Ala7 and Arg8 of H3K9ac form hydrogen bond interactions with AF9 residues Leu108, Leu106, Gly80, and Asp103, respectively. Replacement of Arg8 with an alanine reduced the binding affinity by ~ 200-fold. The binding affinity of H3K9ac to AF9 and ENL were determined with *K*_d_ values of 5 μM and 57 μM [225] (Table 3), respectively.

Peptidic and small molecule inhibitors of ENL YEATS have been reported [200–202]. Peptidic inhibitors XL-07i and XL-13m (Fig. 13h) inhibited ENL-H3K9cr with IC_50_s of 1.3 and 0.56 μM, respectively. The structure of AF9 YEATS in complex with XL-07i has been determined (PDB: 5YYF). XL-07i occupies the H3K9ac binding pocket in YEATS with multiple hydrogen bond and hydrophobic interactions (Fig. 13b, c). In addition, π-π stacking interactions between the furan-2-carbonamide group of XL-07i sandwiched in between Phe59 and Tyr58 seem to strengthen the binding. Additionally, the benzyloxycarbonyl group of XL-07i also has favorable interactions with His107 and His111.

A structure-based approach led to the discovery of a series of benzimidazole inhibitors of ENL YEATS, with SGC-iMLLT (compound 92, Fig. 13h) exhibiting an IC_50_ of 260 nM [200]. The inhibitor was found to suppress expression of leukemia relevant genes such as Myc. The crystal structure of the protein–inhibitor complex (PDB: 6HT1) reveals that the imidazole ring of SGC-iMLLT interacts with Ser76 and the amide is sandwiched between residues Phe59 and Tyr78 with π-π stacking interactions. The amide also acts as a hydrogen bond donor to interact with the hydroxyl group of Ser58. The indazole moiety of the inhibitor is located in a hydrophobic pocket formed by His56, Phe81, Gly80, Ala79, and Tyr78. There is a hydrogen bond between the N-2 atom of the indazole and His56. The 2-methylpyrrolidinyl group of SGC-iMLLT also strengthen the binding by forming a hydrogen bond with Glu75. A high-throughput screening identified a series of piperazine-urea compounds as inhibitors of ENL YEATS [203, 204] with the best compound **1** having an IC_50_ of 3.7 μM (Fig. 13h). Its binding structure has been determined (PDB: 6T1I), showing its piperazine-urea group mimics K9ac (Fig. 13f, g). The 2-methoxyl-pyridine moiety has favorable interactions with His56. In addition, the inhibitor forms hydrogen bonds with ENL residues Ser58 and Tyr78.

## Conclusion and perspectives

MLL1 with 3,696 amino acid residues is an important transcription factor as well as H3K4 methyltransferase. It is a master regulator for transcription of important genes during embryonic development and hematopoiesis, such as clustered Hox genes. MLL1 is essential for development, while it is largely dispensable in matured cells. Dysregulation of MLL1 in these cells leads to constitutive or over-expression of certain Hox genes (e.g., HoxA9) and eventually leukemia initiation. Chromosome translocations involving MLL1 cause ~ 75% of acute leukemia in infants and 5–10% in children and adults with a poor prognosis. Less toxic, targeted therapeutics against onco-MLL1 are needed. Onco-MLL1 consists of the N-terminal DNA-interacting domains of MLL1 fused with one of > 70 fusion partners, among which transcription cofactors AF4, AF9 and its paralog ENL, and ELL are the most frequent. WT- and onco-MLL1 have numerous PPIs, which play critical roles in regulating gene expression in normal physiology and leukemia. Moreover, WT-MLL1 has been found to be essential for MLL1-r leukemia. Therefore, PPIs involving MLL1 as well as its fusion partners are potential drug targets.

Rigorous biological, biochemical and structural studies of such PPIs have been performed to understand their structures, structure–function relationships and the mechanisms for activating gene transcription as well as leukemic transformation. These studies have also revealed and validated that many of these PPIs are promising drug targets for MLL1-r leukemia, such as those in the Menin–MLL1-LEDGF complex and SEC. However, more investigations are needed for the biological functions as well as drug target identification and validation of other PPIs. There are still debates and therefore, a need to address whether MLL1′s H3K4 methyltransferase (SET domain) activity is critical to its biological functions in normal physiology and MLL1-r leukemia. Switching interactions of MLL1′s PHD3-BRD between H3K4-Me3 and Cyp33 have been found to be of importance in gene expression regulation of MLL1, while more in-depth biological studies, particularly in the context of MLL-r leukemia, and pharmacological inhibition of such PPIs are anticipated. Moreover, functions of MLL1′s other PHD domains are poorly understood.

Comparatively, medicinal chemistry progress targeting these PPIs has been considerably falling behind. To date, potent drug-like inhibitors have been discovered and developed for only two cases, i.e., the MLL1-Menin and MLL1-WDR5 interactions. Cellularly expressed peptides or proteins, small peptides, or peptidomimetic compounds were commonly used as an inhibitor for biological investigations. However, these protein or peptide-based inhibitors have poor cell permeability and metabolic stability. Drug-like small molecule inhibitors are desirable, but it is generally difficult for a small molecule to disrupt a PPI with large and shallow surface interactions. However, the MLL1-Menin and -WDR5 interactions are not a typical PPI. Rather, they are (small) MLL1 peptide-protein interactions (Fig. 2a, 7a), similar to a druggable protein such as an enzyme (e.g., kinase) with a peptide substrate. Finding a small molecule inhibitor is more feasible for this kind of PPIs. Fortunately, several other critical PPIs involving MLL1 and its fusion proteins fall into this category, including MLL1-LEDGF and -H3K4Me3, AF9/ENL-AF4/AFF4/DOT1L and AF4/AFF4-cyclin T1. Medicinal chemistry studies targeting these PPIs could be fruitful in the perspectives of drug discovery targeting MLL1-r leukemia. Availability of the X-ray or NMR structures and biochemical assays of these PPIs could further facilitate this process.

Small molecule inhibitors of the MLL1-Menin interaction have shown excellent activity against MLL1-r leukemia in cells and animal models [16, 49, 50, 52–54, 59, 61, 63], which are generally correlated with their biochemical activities. Many inhibitors also possess good pharmacokinetic profiles, among which KO-539 and SNDX 5613 have entered into Phase I clinical trials for AML (Table 2) [16]. On the other hand, despite high biochemical potencies, the small molecule inhibitors of the MLL1-WDR5 interaction showed less or inconsistent cellular anti-leukemia activities, presumably due to complex functions of MLL1′s H3K4 methyltransferase activity or off-target effects.

Potential toxicities related to inhibiting the PPIs involving MLL1 and its fusion partners are a concern. Since MLL1 is critical to embryonic development and hematopoiesis, but largely dispensable in matured cells including hematopoiesis in adult mice, inhibition of these PPIs in the context of targeting MLL1-r leukemia appears to be selective with a likely high therapeutic index. This has been observed for the inhibitors of MLL1-Menin. However, MLL1 plays a role in self-renewal of hematopoietic stem cells in bone marrow and its conditional knockout in adult mice showed some defects [227]. Moreover, Menin has been reported to be a tumor suppressor and its mutation is involved in inherited tumor syndrome multiple endocrine neoplasia type 1 [104]. Similarly, germline knockout of Menin is embryonic lethal in mice [228] and its conditional knockout in adult mice could also affect hematopoiesis and myeloid transformation [113]. Potential toxicities of these compounds that bind to Menin and inhibit the MLL1-Menin interaction need to be closely observed during clinical trials. Similar scenarios exist for other PPIs involving MLL1 (e.g., MLL1-LEDGF) as well as those in SEC.

In conclusion, PPIs involving MLL1 and its fusion partners play critical roles in gene expression regulation during development, while they are largely dispensable in adults. However, these PPIs have been found to be essential for MLL1-r leukemia, making them potential drug targets for intervention. Pharmacological inhibition of several such PPIs has been pursued. Inhibitors of MLL1-Menin have shown promising preclinical results and entered clinical trials against AML. Further drug discovery efforts targeting other PPIs including biological and medicinal chemistry investigation are warranted.

## Data Availability

Not applicable.

## Abbreviations

MLL1/MLL: Mixed lineage leukemia 1
Onco-MLL1: Oncogenic fusion MLL1
MLL1-r: MLL1-rearranged
ALL: Acute lymphoblastic leukemia
AML: Acute myeloid leukemia
PPIs: Protein–protein interactions
ATH: AT-hooks
H3K4: Histone-H3 lysine-4
KMT: Lysine methyltransferase
MBM: Menin-binding motif
LEDGF: Lens epithelium-derived growth factor
LBD: LEDGF-binding domain
NLS: Nuclear localization signal
PHD: Plant homology domains
BRD: Bromodomain
TCS: Taspase 1 cleavage site
PAFc: Polymerase-associated factor complex
TAD: Transactivation domain
WT: Wild type
FYRN: Phe/Tyr-rich N-terminal
FYRC: Phe/Tyr-rich C-terminal
WDR5: WD repeat protein 5
Win: WDR5 interaction motif
SET: Su(Var)3–9, enhancer-of-zeste, trithorax domain
MLL1-N: MLL1 N-terminal fragment
MLL1-C: MLL1 C-terminal fragment
RbBP5: Retinoblastoma binding protein 5
ASH2L: Set1/Ash2 histone methyltransferase complex subunit ASH2-like
DPY30: Protein dpy-30 homolog
AF4: ALL-1 fused chromosome 4
SEC: Superelongation complex
Pol II: RNA polymerase II
HTS: High throughput screening
SAR: Structure–activity relationship
MBM: Menin–MLL1 binding motif
IBD: Integrase binding domain
HIV: Human immunodeficiency virus
HMT: Histone methyltransferase
SPR: Surface plasmon resonance
AHD: ANC1 homology domain
CBX8: Chromobox homolog 8
PRC1: Polycomb repressive complex 1
PK: Pharmacokinetics
PDX: Patient-derived xenograft
NPM1: Nucleophosmin 1
PWWP: Pro-Try-Try-Pro
CR: Charged regions
IBM: IBD-binding motifs
H3K36-Me3: Trimethylated H3K36
RRM: RNA recognition motif
HDAC1: Histone deacetylase 1
CBP: CAMP-response element binding protein (CREB)-binding protein
HAT: Histone acetyltransferase
ABM: ASH2L binding motif
WBM: WDR5-binding motif
P-TEFb: Positive transcription elongation factor
CHD: C-terminal homology domain
CDK9: Cyclin-dependent kinase 9
TAR: Transactivating response region
YEATS: Yaf9, ENL, AF9, Taf14, and Sas5
BCoR: BCL-6 corepressor
